# Interleukin-10 contributes to reservoir establishment and persistence in SIV-infected macaques treated with antiretroviral therapy

**DOI:** 10.1172/JCI155251

**Published:** 2022-04-15

**Authors:** Justin Harper, Susan P. Ribeiro, Chi Ngai Chan, Malika Aid, Claire Deleage, Luca Micci, Maria Pino, Barbara Cervasi, Gopalan Raghunathan, Eric Rimmer, Gulesi Ayanoglu, Guoxin Wu, Neeta Shenvi, Richard J.O. Barnard, Gregory Q. Del Prete, Kathleen Busman-Sahay, Guido Silvestri, Deanna A. Kulpa, Steven E. Bosinger, Kirk A. Easley, Bonnie J. Howell, Dan Gorman, Daria J. Hazuda, Jacob D. Estes, Rafick-Pierre Sekaly, Mirko Paiardini

**Affiliations:** 1 Division of Microbiology and Immunology, Yerkes National Primate Research Center, Emory University, Atlanta, Georgia, USA.; 2 Department of Pathology, Case Western Reserve University, Cleveland, Ohio, USA.; 3 Vaccine and Gene Therapy Institute, Oregon Health & Science University, Beaverton, Oregon, USA.; 4 Center for Virology and Vaccine Research, Beth Israel Deaconess Medical Center, Boston, Massachusetts, USA.; 5 AIDS and Cancer Virus Program, Leidos Biomedical Research, Frederick National Laboratory for Cancer Research, National Cancer Institute, NIH, Frederick, Maryland, USA.; 6 Department of Discovery Oncology, Merck & Co., Inc., Boston, Massachusetts, USA.; 7 Flow Cytometry Core, Emory Vaccine Center, Emory University, Atlanta, Georgia, USA.; 8 Department of Discovery Biologics and; 9 Department of Pharmacokinetics, Pharmacodynamics and Drug Metabolism, Merck & Co., Inc., South San Francisco, California, USA.; 10 Department of Infectious Disease, Merck & Co., Inc., West Point, Pennsylvania, USA.; 11 Department of Biostatistics and Bioinformatics, Rollins School of Public Health, Emory University, Atlanta, Georgia, USA.; 12 Division of Pathobiology and Immunology, Oregon National Primate Research Center, Oregon Health & Science University, Beaverton, Oregon, USA.; 13 Department of Pathology and Laboratory Medicine, Emory University School of Medicine, Atlanta, Georgia, USA.

**Keywords:** AIDS/HIV, Immunology, Immunotherapy, T cells

## Abstract

Interleukin-10 (IL-10) is an immunosuppressive cytokine that signals through STAT3 to regulate T follicular helper (Tfh) cell differentiation and germinal center formation. In SIV-infected macaques, levels of IL-10 in plasma and lymph nodes (LNs) were induced by infection and not normalized with antiretroviral therapy (ART). During chronic infection, plasma IL-10 and transcriptomic signatures of IL-10 signaling were correlated with the cell-associated SIV-DNA content within LN CD4^+^ memory subsets, including Tfh cells, and predicted the frequency of CD4^+^ Tfh cells and their cell-associated SIV-DNA content during ART, respectively. In ART-treated rhesus macaques, cells harboring SIV-DNA by DNAscope were preferentially found in the LN B cell follicle in proximity to IL-10. Finally, we demonstrated that the in vivo neutralization of soluble IL-10 in ART-treated, SIV-infected macaques reduced B cell follicle maintenance and, by extension, LN memory CD4^+^ T cells, including Tfh cells and those expressing PD-1 and CTLA-4. Thus, these data support a role for IL-10 in maintaining a pool of target cells in lymphoid tissue that serve as a niche for viral persistence. Targeting IL-10 signaling to impair CD4^+^ T cell survival and improve antiviral immune responses may represent a novel approach to limit viral persistence in ART-suppressed people living with HIV.

## Graphical abstract

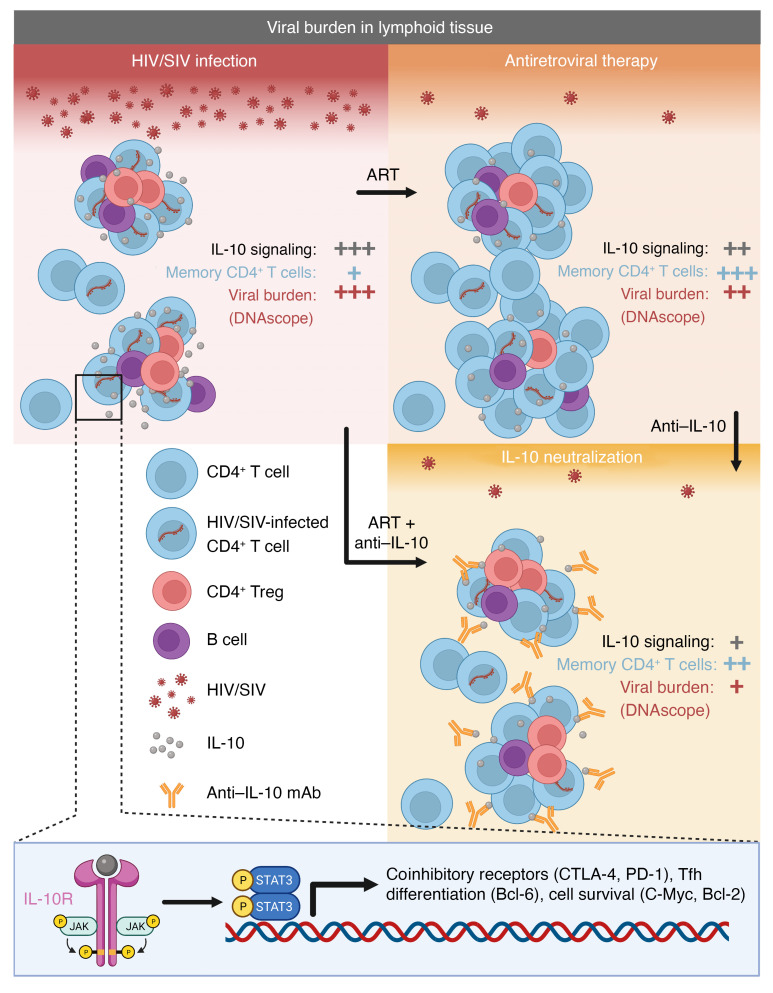

## Introduction

While the optimization of antiretroviral therapy (ART) has prevented AIDS and reduced HIV-related morbidities and mortality for the majority of people living with HIV (PLWH), a therapeutic regimen capable of sustaining prolonged, immune-mediated viral remission without ART does not currently exist. An obstacle to achieving viral remission is the presence of a “reservoir” of long-lived, HIV-infected CD4^+^ T cells, which variably harbor replication-competent virus during ART and are responsible for viral recrudescence upon ART cessation (1–3). The mechanisms that control the establishment and maintenance of HIV reservoirs are unknown, thus impeding the design of selective strategies limiting HIV persistence. Recent findings indicate that T follicular helper (Tfh) CD4^+^ T cells, which express programmed cell death protein 1 (PD-1) and reside in B cell follicles (BCFs) of secondary lymphoid organs, are infected in viremic PLWH and constitute a compartment in which replication-competent HIV/SIV persists during ART (4, 5). Furthermore, SIV replication is restricted to CD4^+^ Tfh cells in the presence of potent SIV-specific CD8^+^ T cell responses in elite controller rhesus macaques (RMs) (6). In addition to Tfh cells, cytotoxic T lymphocyte–associated protein 4–positive (CTLA-4^+^) PD-1–negative CD4^+^ T cells, which possess phenotypic and functional features of regulatory T cells (Tregs), are also key contributors to viral persistence during ART (7, 8). Thus, cells with a central role in regulating T cell homeostasis and immune responses are targeted by the virus to maintain viral persistence.

Interleukin-10 (IL-10) is a critical component of the immunosuppressive network required to dampen the proinflammatory responses activated by the immune system after an encounter with a pathogen, and has been shown to be rapidly induced following SIV infection (9, 10). IL-10 is produced by several innate and adaptive immune cells (monocytes/macrophages, dendritic cells, B cells, Tr1 cells) and binds to the IL-10 receptor (IL-10R) on macrophages and dendritic cells to reduce antigen presentation and trigger T cell anergy (11–13). Furthermore, IL-10 signaling inhibits the production of Th1 cytokines, such as IL-12 and interferon-γ (IFN-γ), whereas it stimulates the production of Th2 cytokines, including IL-4, IL-15, and IL-13 (14, 15). In this antiinflammatory environment in response to pathogen-induced inflammation, elevated or even physiological levels of IL-10 can negatively impact T cell effector function and lead to a status of immunosenescence, which favors the persistence of viral infections (16–19). In mouse models, lymphocytic choriomeningitis virus infection results in a rapid induction of IL-10, which operates on immunoinhibitory pathways distinct from the PD-L1 axis (20), to specifically suppress IFN-γ responses from CD4^+^ T cells (21). Furthermore, the in vivo blockade of IL-10Rα and its genetic deficiency in mice have also shown promise in restoring effective antiviral responses to promote pathogen clearance (16, 22, 23).

Analogous to observations with other chronic viral infections, levels of IL-10, both in plasma and by mRNA levels in peripheral blood mononuclear cells (PBMCs), correlate with viral loads in viremic PLWH and attenuate with ART (17, 24, 25). During chronic HIV infection, IL-10 production correlates with heightened PD-1 expression on monocytes (10) and constrains the proliferative capacity and the cytokine production (i.e., IFN-γ) of HIV-specific CD4^+^ T cells as deduced from the ex vivo blockade of IL-10Rα (17, 26); however, the in vivo administration of recombinant IL-10 does not impact plasma viral loads or CD4^+^ T cell counts (27). Key IL-10–producing cells, such as B cells and Tregs, are present at high frequencies in lymphoid tissue BCFs, wherein CD4^+^ Tfh cells serve as a key cellular niche for HIV/SIV persistence (4, 5, 7, 28). As IL-10 signals through signal transducer and activator of transcription 3 (STAT3) (29), a critical regulator of Tfh cell differentiation and BCF formation and maintenance (30–33), we hypothesized a nonspecific role for IL-10 in promoting the survival and differentiation of cellular subsets responsible for HIV/SIV persistence within lymphoid tissues. To address these questions, using SIV-infected RMs we quantified levels of IL-10 in plasma and lymphoid tissues, and transcriptomic signatures of IL-10 signaling in PBMCs, which were correlated against parameters of disease progression and viral persistence during chronic infection and following ART suppression. To validate the causality between IL-10 and the maintenance of lymphoid tissue CD4^+^ Tfh cells, we performed a pilot study in which a neutralizing anti–IL-10 monoclonal antibody (mAb) was administered in vivo to SIV-infected, ART-treated RMs. Altogether, our findings support a role for IL-10 in favoring the maintenance of cellular reservoirs of viral persistence during ART and provide a rationale for therapeutic approaches targeting IL-10 signaling as a strategy for HIV remission.

## Results

### IL-10 during chronic infection correlates with signatures of T cell exhaustion and survival.

**Figure 1:**
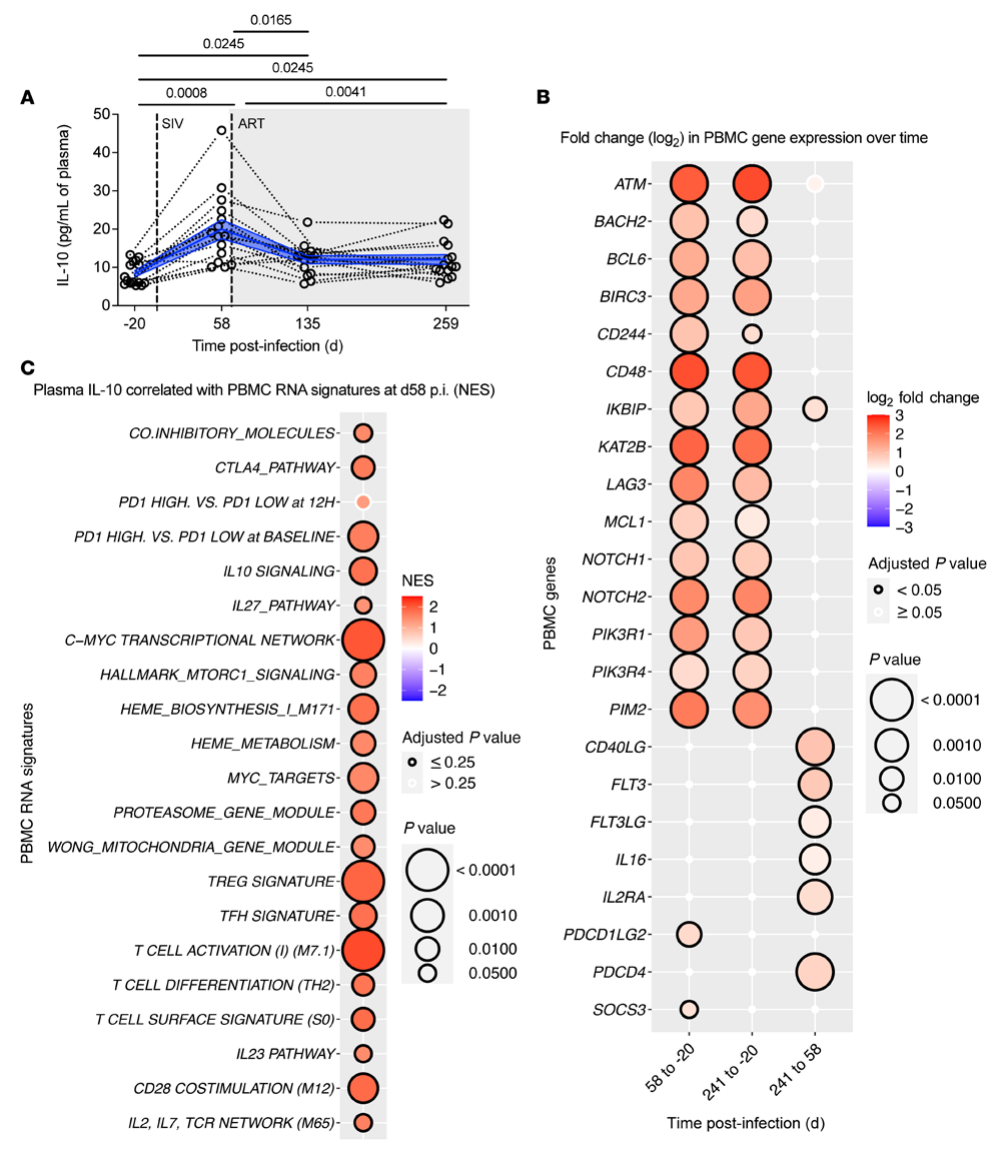
IL-10 during chronic infection correlates with signatures of T cell exhaustion and survival. (**A**) Plasma IL-10 levels (pg/mL; 0.2 pg/mL limit of detection) were assessed by an ultrasensitive sandwich immunoassay at pre-infection (d–20 p.i.), at chronic SIV infection (d58 p.i.), and during suppressive ART (d135 and d259 p.i.; *n =* 15). Ongoing ART is given by the gray-shaded background, and data from individual RMs are represented by tethered, open black circles with averaged data presented as mean (blue line) ± SEM (blue filled area). Data were analyzed with 2-sided, 1-way ANOVA using Holm-Šidák correction for multiple comparisons between all time points. (**B**) In PBMCs (*n =* 15), mRNA transcripts of genes associated with activation, Tfh cell homeostasis, and immune checkpoint receptor (ICR) expression were measured by RNA-Seq (annotated at left). The log_2_ fold change in abundance over time (i.e., between d–20, d58, and d241 p.i., as indicated below) is indicated by the bidirectional color-coded heatmap. Point size corresponds to the log_10_-transformed *P* value with a threshold for highly significant values (*P <* 0.0001), and significant adjusted *P* values (*P <* 0.05) are indicated by a black border (legend at right). (**C**) Transcriptomic signatures of exhaustion, survival, and activation were measured by RNA-Seq in PBMCs (*n =* 15) at chronic infection (as annotated at left) and were correlated against levels of plasma IL-10 (pg/mL) during chronic infection. Normalized enrichment scores (NES) are represented by a bidirectional color-coded heatmap. Point size corresponds to the log_10_-transformed *P* value with a threshold for highly significant values (*P <* 0.001), and significant adjusted *P* values (*P <* 0.25) are indicated by a black border (as shown at right). (**B** and **C**) Statistical analyses were calculated by gene set enrichment analysis (GSEA).

To corroborate observations in PLWH (17, 24, 25), we monitored the levels of plasma IL-10 using an ultrasensitive sandwich immunoassay in 15 SIVmac_239_-infected RMs that initiated a 5-drug ART regimen (raltegravir, emtricitabine, tenofovir, darunavir, ritonavir) at day 60 post-infection (d60 p.i.) (ref. 34 and Supplemental Table 1; supplemental material available online with this article; https://doi.org/10.1172/JCI155251DS1). Plasma IL-10 during chronic infection (d58 p.i.) was elevated relative to pre-infection (d–20; 19.96 ± 2.481 pg/mL vs. 8.32 ± 0.7849 pg/mL; Figure 1A). Following ART initiation, plasma IL-10 rapidly decreased (12.01 ± 1.041 pg/mL at d135 p.i.) and stabilized at levels still higher (12.06 ± 1.28 pg/mL at d259 p.i.) as compared with pre-infection (Figure 1A). High levels of plasma IL-10 in rhesus macaque RGv10 during chronic infection (45.8 pg/mL; 3.44-fold change) were identified as a statistical outlier by Grubbs’s test (α = 0.05), whose inclusion does not impact the significance of this or subsequent correlative analyses. Consistent with plasma IL-10, we found that the expression of multiple IL-10–regulated transcripts was strongly stimulated by SIV infection (d58 vs. d–20 p.i.; Figure 1B), including B cell lymphoma 6 protein (*BCL-6*), the master transcriptional regulator of Tfh cell differentiation (35, 36); induced myeloid leukemia cell differentiation protein (*MCL1*), a BCL-2 family antiapoptotic factor critical for the survival of activated T cells (37); and immune checkpoint receptors (ICRs; e.g., *LAG3*), which reduce effector T cell function and identify CD4^+^ T cells enriched in HIV reservoir (7, 38–40). Expression of these transcripts remained elevated during ART as compared with pre-infection (d241 vs. d–20 p.i.), and during ART a subset of transcripts were continually expressed at levels comparable to those observed in chronic infection (d241 vs. d58 p.i.; Figure 1B). To validate an impact on a protein level, in vitro IL-10 stimulation was performed using PBMCs from uninfected RMs, which resulted in the upregulation of phosphorylated STAT3 (p-STAT3) (Supplemental Figure 1A), the transcription factor directly downstream of IL-10 signaling that participates in regulating T cell survival and homeostatic proliferation (10, 33, 41, 42); BCL-6 (Supplemental Figure 1B); and ICRs such as CTLA-4 (Supplemental Figure 1C). Consistent with their expression being induced by IL-10 signaling, these markers were not upregulated when IL-10 stimulation was blocked by the presence of an anti–IL-10 mAb (Supplemental Figure 1, A–C). To explore the possibility of a link between IL-10 and viral persistence, levels of plasma IL-10 during chronic infection (d58 p.i.) were correlated with PBMC transcriptomic signatures at the same time point (Figure 1C). Plasma IL-10 correlated with higher expression of transcriptomic signatures related to promoting cell exhaustion including the *IL-10*, *IL-27*, *CTLA-4*, and *PD-1* pathways, of which *IL-27* is a known inducer of IL-10 (43–46). Plasma IL-10 also correlated with the transcriptomic signatures promoting cell growth and survival, including multiple transcripts belonging to C-Myc (*BIRC5*, *CKS2*, *TOP2A*, *CDCA7*), mTOR (*PIK3R3*, *FADS1*, *FADS2*, *PLK*), and heme metabolism (*ALAS2*, *ALAD*) pathways. Furthermore, plasma IL-10 correlated with transcriptomic signatures promoting the differentiation of SIV-harboring CD4^+^ T cell subsets, such as Tfh cells and Tregs (Figure 1C and refs. 5, 7). Given the downstream signaling targets of IL-10, which is itself induced by SIV infection and not fully normalized with ART, these data suggest that IL-10 may in turn regulate viral seeding and persistence.

### Plasma IL-10 during chronic infection correlates with lymphoid SIV-DNA content.

**Figure 2:**
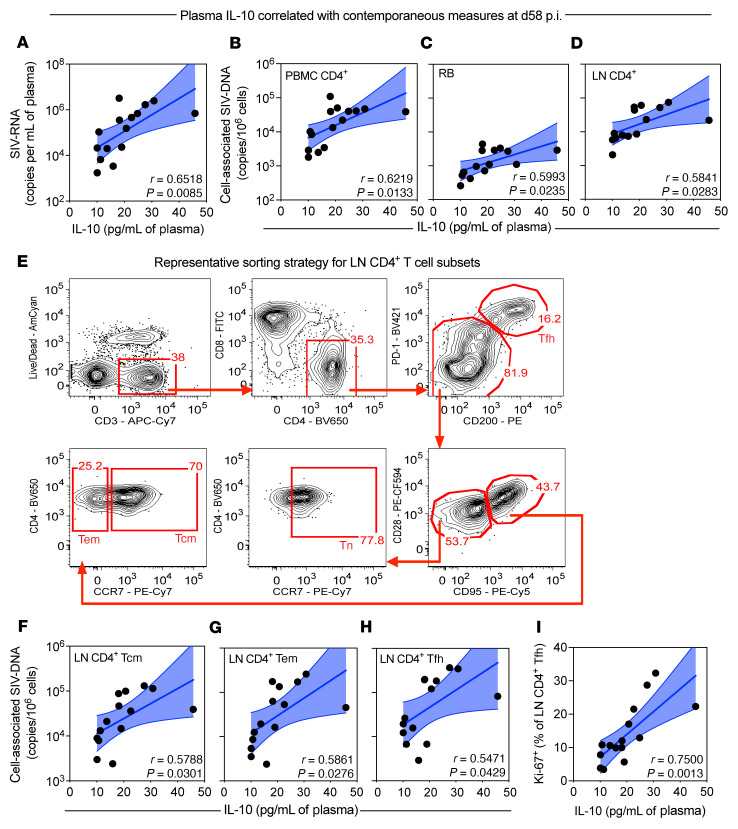
Plasma IL-10 during chronic infection correlates with lymphoid SIV-DNA content. (**A**–**D**) The concentration of plasma IL-10 (pg/mL; 0.2 pg/mL limit of detection) was measured by an ultrasensitive sandwich immunoassay during chronic infection (d58 p.i.) and was correlated against the plasma viral load (log_10_ SIV-RNA copies/mL; *n =* 15) (**A**) and the cell-associated SIV-DNA content (log_10_ copies per 10^6^ cells) by RT-qPCR in PBMC CD4^+^ T cells (*n =* 15) (**B**), rectal biopsy (RB) mononuclear cells (*n =* 14) (**C**), and lymph node (LN) CD4^+^ T cells (*n =* 14) (**D**). (**E**) LN mononuclear cells underwent FACS for memory CD4^+^ T cell subsets as follows: central memory (Tcm: PD-1^dim^CD200^–^CD95^+^CCR7^+^), effector memory (Tem: PD-1^dim^CD200^–^CD95^+^CCR7^–^), T follicular helper (Tfh; CD200^+^PD-1^hi^), and naive (Tn: PD-1^dim^CD200^–^CD95^–^CCR7^+^). RLm12 at d58 p.i. is shown as a representative sorting strategy pre-gated on singlet lymphocytes (1 of 14 unique sorts with 20,000 pre-sort events). (**F**–**I**) Plasma IL-10 (pg/mL) during chronic infection was correlated against the cell-associated SIV-DNA content (log_10_ copies per 10^6^ cells) in LN CD4^+^ Tcm (**F**), LN CD4^+^ Tem (**G**), and LN CD4^+^ Tfh cells (**H**) (*n =* 14 each); and the percentage of proliferating (Ki-67^+^) LN CD4^+^ Tfh cells by flow cytometry (*n =* 15) (**I**). (**A**–**D** and **F**–**I**) Data from individual macaques are represented as black circles and are overlaid with a simple linear regression (solid blue line) with a 95% confidence interval (blue fill). All correlations were performed using a 2-sided Pearson’s correlation coefficient.

To further characterize the role of IL-10 in regulating SIV persistence in vivo, we correlated the ultrasensitive levels of plasma IL-10 (Figure 1A) with measures of disease progression and viral content during chronic infection. SIV-induced levels of plasma IL-10 correlated with parameters of disease progression including plasma viremia (Figure 2A), the depletion of CD4^+^ T cells in blood (Supplemental Figure 2A), and levels of IFN-γ–induced protein 10 (IP-10; Supplemental Figure 2B), an early soluble activation marker strongly associated with disease progression (47). These correlations suggest that IL-10 expression is induced as a counter-regulatory mechanism to balance the excessive inflammation during chronic infection. Levels of plasma IL-10 during chronic infection correlated with the frequencies of cell-associated SIV-DNA content in PBMC CD4^+^ T cells, rectal mucosa, and lymph node (LN) CD4^+^ T cells (Figure 2, B–D). As IL-10–stimulated pathways impact T cell differentiation (Figure 1C), we measured the viral content in the following LN CD4^+^ T cell subsets: central memory (Tcm: PD-1^dim^CD200^–^CD95^+^CCR7^+^), effector memory (Tem: PD-1^dim^CD200^–^CD95^+^CCR7^–^), and Tfh (CD200^+^PD-1^hi^) (representative gating shown in Figure 2E). Plasma IL-10 correlated with cell-associated SIV-DNA content in the Tcm, Tem, and Tfh subsets (Figure 2, F–H), as well as with the frequency of proliferating (Ki-67^+^) CD4^+^ Tfh cells (Figure 2I). Notably, the described correlations were specific for IL-10 and were not found for several other soluble markers of inflammation (sCD14, sCD163, CRP, neopterin, and LPS) apart from IP-10 and plasma viremia (Supplemental Table 2), which were further statistically interrogated and determined to not exhibit multicollinearity with IL-10 based on their low variance inflation factors (<4) and condition indices (<30; Supplemental Table 3). As a caveat, these correlative analyses for cell-associated SIV-DNA do not exclude cells harboring defective proviruses; however, others have reported that SIV genomes are more intact than those observed in PLWH (2, 48, 49), and measures of cell-associated HIV-DNA are correlated with values of replication-competent virus by an intact proviral DNA assay and quantitative viral outgrowth assay (QVOA) (50, 51). Therefore, as plasma levels of IL-10 correlate with the expansion of Tfh cells during chronic infection (52, 53) and their respective SIV-DNA content, these data support a role for IL-10 in maintaining a pool of target cells in lymphoid tissue that serve as a niche for viral persistence.

### Lymphoid IL-10^+^ cells reside in close spatial proximity to cells harboring SIV-DNA.

**Figure 3:**
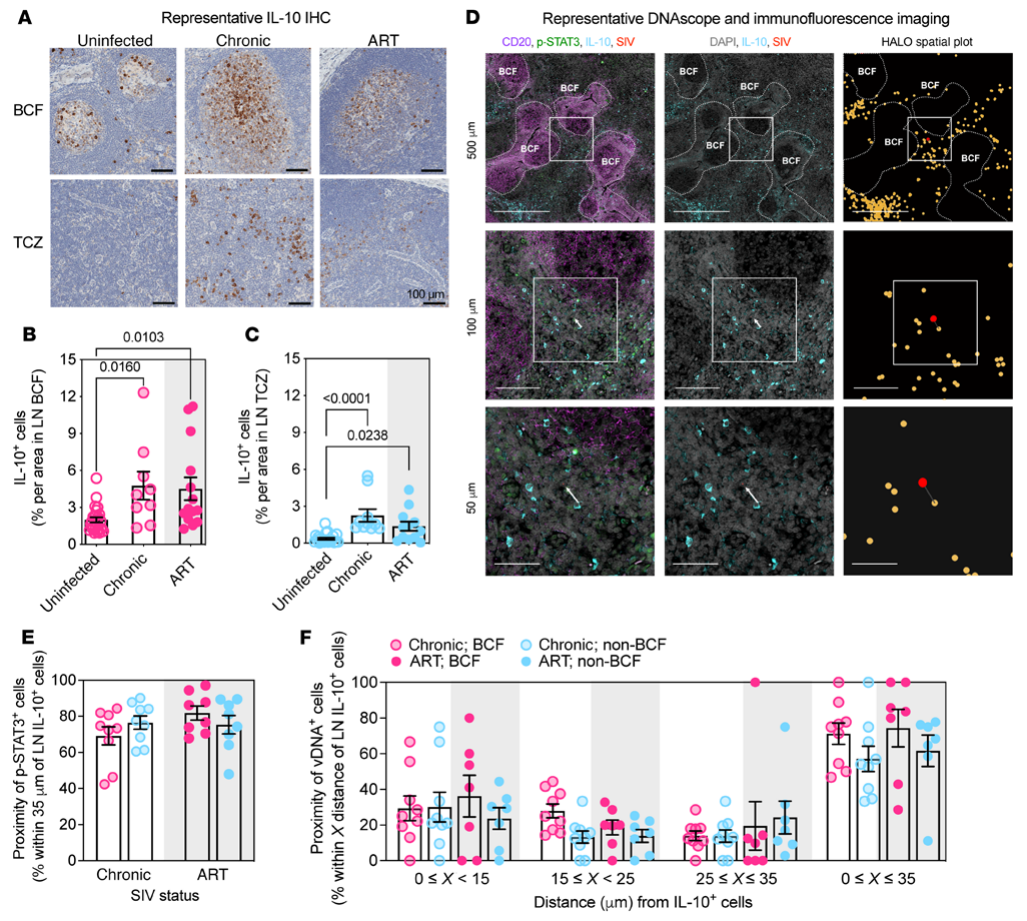
Lymphoid IL-10^+^ cells reside in close spatial proximity to cells harboring SIV-DNA. (**A**) IHC was performed for IL-10 in the lymphoid BCF and T cell zone (TCZ) before SIV infection, during chronic SIV infection, or following ART initiated during chronic infection (Supplemental Table 4). Representative IHC shown is rhesus macaque ZI26 at pre-infection, 18 weeks p.i., and 30 weeks p.i. following 12 weeks on ART (3 of 56 unique). Scale bars: 100 μm. (**B** and **C**) Percentage of IL-10^+^ cells by area was determined within the BCF (pink; uninfected, *n =* 25; chronic, *n =* 9; ART, *n =* 14) (**B**) and TCZ (blue; uninfected, *n =* 23; chronic, *n =* 10; ART, *n =* 12) (**C**), analyzed by unmatched 1-way ANOVA with Tukey’s correction. (**D**) DNAscope in situ hybridization for cell-associated viral DNA (vDNA; red) was performed in combination with immunofluorescence imaging to visualize cells costaining with IL-10 (cyan) and p-STAT3 (green) in the LN BCF and non-BCF (i.e., TCZ and medullary cords; RGv10 at 8 months of ART; 1 of 17 unique). Scale bars: 500, 100, 50 μm. Cell numbers were determined with DAPI nuclear stain (gray). The spatial proximity map shows the location of vDNA^+^ (red) and IL-10^+^ (yellow) cells as detected and assigned by HALO software (Indica Labs v3.0.311.405) with the nearest neighbor indicated by the proximity line in gray. (**E** and **F**) The frequency of p-STAT3^+^ (chronic, *n =* 9; ART, *n =* 8) (**E**) and vDNA^+^ (chronic, *n =* 9; ART, *n =* 7) (**F**) cells was calculated based on their spatial proximity to the nearest IL-10^+^ cell in the BCF and non-BCF during chronic infection and with ART of variable duration (Supplemental Table 4). (**B**, **C**, **E**, and **F**) Gray shading represents ART, and individual RMs are represented as open (chronic) or filled (ART) circles color-coded per anatomical localization (pink, BCF; blue, non-BCF) with averaged data presented as mean ± SEM (black) that were analyzed with a 1-way (**E**) or 2-way (**F**) mixed-effects model with Tukey’s correction.

As peripheral levels of IL-10 were correlated with the tissue cell-associated SIV-DNA content during chronic infection, we next sought to determine whether in situ levels of IL-10 in LNs were similarly tied to the extent of infection. By immunohistochemistry (IHC) we quantified the levels of IL-10^+^ cells in the LN of uninfected, chronically infected, and ART-suppressed RMs from cross-sectional, historical cohorts (Figure 3A and Supplemental Table 4). Consistent with the results in plasma, the percentage area of LN BCF that stained for IL-10 was higher in chronically infected RMs as compared with uninfected (*n =* 9, 4.765% ± 1.137% area of IL-10^+^ cells, vs. *n =* 25, 1.986% ± 0.2073% area of IL-10^+^ cells), and remained elevated in ART-suppressed RMs (*n =* 14, 4.512% ± 0.9277%; Figure 3B). Similar results were found in the LN T cell zone (TCZ), with IL-10 higher in chronically infected RMs compared with uninfected RMs (*n =* 10, 2.255% ± 0.5124% area of IL-10^+^ cells, vs. *n =* 23, 0.3627% ± 0.07951% area of IL-10^+^ cells), which failed to fully normalize in ART-suppressed RMs (*n =* 12, 1.379% ± 0.3763 area of IL-10^+^ cells; Figure 3C). As ART likewise fails to normalize LN levels of IL-10, we hypothesized that continuous IL-10 signaling could favor viral persistence.

To determine whether IL-10 supports viral persistence within lymphoid tissues, critical sites for HIV and SIV reservoir maintenance (3), we detected cell-associated viral DNA (vDNA) via an in situ DNAscope hybridization assay in conjunction with immunofluorescence microscopy (28). To determine the role of IL-10 during both early and prolonged ART, DNAscope analyses were performed at different time points after ART initiation, including in 2 of 8 RMs that had not yet attained complete viral control (<60 copies/mL; Supplemental Table 4). As shown in the representative images in Figure 3D and quantified in Figure 3E, the majority of follicular (“BCF”) and non-follicular (“non-BCF,” including the TCZ and medullary cords) p-STAT3^+^ cells were consistently in close proximity (≤35 μm) to IL-10–producing cells both during chronic infection (*n =* 9) and during ART (*n =* 8), confirming that mere spatial proximity is an approximate, yet sufficient, surrogate of IL-10–mediated p-STAT3 signaling. A proximity threshold of 35 μm (i.e., approximately 3 cell diameters) was chosen to capture cells likely within recent contact of IL-10^+^ cells based on T cell homing velocities in LNs (54). Consistent with observations of p-STAT3–expressing cells (Figure 3E), the majority of follicular and non-follicular vDNA^+^ cells were colocalized with IL-10–producing cells both during chronic infection (BCF, 71.22% ± 5.96%; non-BCF, 57.09% ± 7.10%; *n =* 9) and during ART (BCF, 74.40% ± 10.52%; non-BCF, 61.67% ± 8.82%; *n =* 7; Figure 3F). As IL-10 signaling correlates with signatures of survival and T cell differentiation, and cells within lymphoid tissue are subject to continuous IL-10 expression despite suppressive ART, these spatial proximity analyses suggest that IL-10 signaling aids in maintaining the pool of cells harboring cell-associated vDNA.

### IL-10 predicts the frequency and SIV-DNA content of LN CD4^+^ Tfh cells during ART.

**Figure 4:**
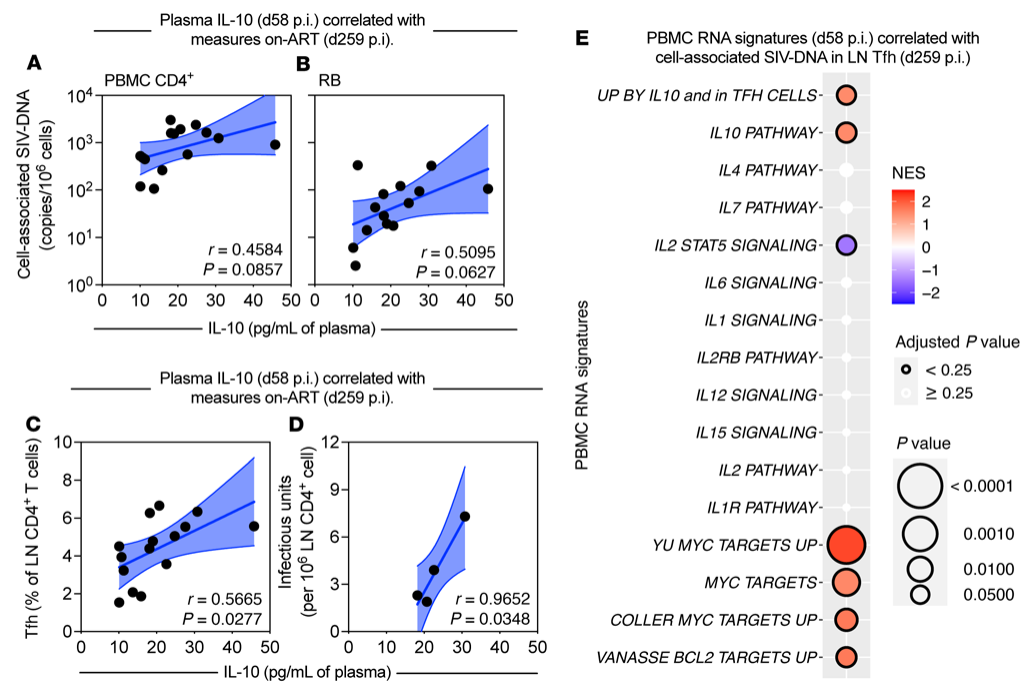
IL-10 predicts the frequency and SIV-DNA content of LN CD4^+^ Tfh cells during ART. (**A**–**D**) Levels of plasma IL-10 (pg/mL; *n =* 15; 0.2 pg/mL limit of detection) were measured by an ultrasensitive sandwich immunoassay in chronic infection (d58 p.i.) and were correlated against the following parameters after 7 months of ART (d259 p.i.): cell-associated SIV-DNA content (log_10_ copies per 10^6^ cells) by RT-qPCR in PBMC CD4^+^ T cells (*n =* 15) (**A**) and rectal biopsy (RB) mononuclear cells (*n =* 14) (**B**); frequency of LN Tfh cells (CXCR5^+^PD-1^hi^) among CD4^+^ T cells by flow cytometry (*n =* 15) (**C**); and, in a subset of RMs (*n =* 4), the infectious units per million total CD4^+^ cells in LNs by QVOA (**D**). Data from individual RMs are represented as black circles and are overlaid with a simple linear regression (solid blue line) with a 95% confidence interval (blue fill). Correlations were performed by 2-sided Pearson’s correlation coefficient. (**E**) In PBMCs from chronic infection, transcriptomic signatures of cytokine signaling, survival, and cell cycling were measured by RNA-Seq and correlated against cell-associated SIV-DNA content in LN CD4^+^ Tfh cells during ART (*n =* 13). Normalized enrichment scores (NES) are represented by a bidirectional color-coded heatmap. Point size corresponds to the log_10_-transformed *P* value with a threshold for highly significant values (*P <* 0.0001), and significant adjusted *P* values (*P <* 0.25) are indicated by a black border (legend at right) as calculated by GSEA.

As our findings highlight IL-10 signaling as a mechanism of cell survival and viral persistence, using our longitudinal cohort (*n =* 15) we investigated whether RMs with higher levels of plasma IL-10 during chronic infection (d58 p.i.) had increased viral content once ART-suppressed (d259 p.i., 7 months of ART). Notably, these analyses included a subset of animals treated with IL-21 following ART initiation (*n =* 7; Supplemental Table 1 and ref. 34) that, in and of itself, did not impact levels of plasma IL-10 or the frequency of LN CD4^+^ Tfh cells (Supplemental Figure 3, A and B). Higher levels of IL-10 (Figure 1A) during chronic infection positively correlated with the cell-associated SIV-DNA content in blood CD4^+^ T cells and rectal mucosa (Figure 4, A and B) as well as with the frequency of LN CD4^+^ Tfh cells (CXCR5^+^PD-1^hi^) following 7 months of ART (Figure 4C). Plasma levels of IL-10 during chronic infection also positively correlated with the frequency of replication-competent virus in LN total CD4^+^ cells during ART, although this analysis was limited to a subset of RMs with detectable viral content by QVOA (*n =* 4; Figure 4D). The capacity of IL-10 to predict these parameters was superior as compared with other soluble inflammatory biomarkers (CRP, LPS, sCD14, sCD163, IP-10, and neopterin; Supplemental Table 5). Furthermore, PBMC transcriptomics signatures of *IL-10* signaling, including pathways for Tfh cell differentiation, cell cycling, and survival, were predictive of the cell-associated SIV-DNA content in LN CD4^+^ Tfh cells during ART, independent of signatures of *IL-2*, *IL-4*, *IL-6*, *IL-7*, and *IL-15* signaling (Figure 4E). These data highlight that the induction of IL-10 following SIV infection is associated with the maintenance of the viral reservoir and the frequency of target cells, including LN CD4^+^ Tfh cells. Collectively, these data support a molecular link between IL-10 expression and residual disease during ART, and highlight IL-10 signaling as a regulator of cellular homeostasis, including target cells that support viral persistence, in blood and tissues.

### IL-10 neutralization inhibits memory CD4^+^ T cell homeostasis in vivo.

**Figure 5:**
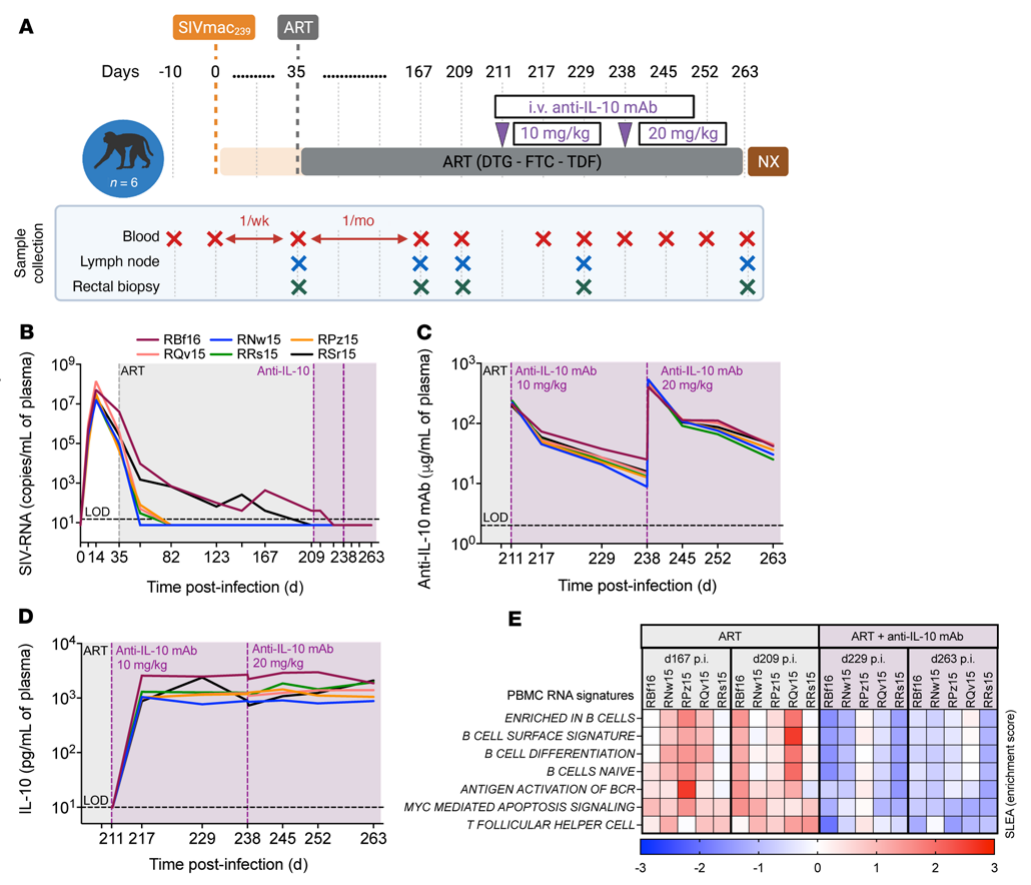
IL-10 neutralization inhibits memory CD4^+^ T cell homeostasis in vivo. (**A**) Six RMs were infected i.v. with SIVmac_239_ and, at d35 p.i., began daily ART. All RMs received an anti–IL-10 mAb at d211 p.i. (10 mg/kg) and at d238 p.i. (20 mg/kg) followed by necropsy (NX) at d263 p.i. Tissue collections, including blood, LN biopsy, and rectal biopsy, are indicated below. (**B**) Longitudinal plasma viral loads (SIV-RNA copies/mL) were determined by RT-qPCR with 6 replicate reactions (15-copies/mL limit of detection indicated by horizontal dashed line; *n =* 6). (**C** and **D**) During the intervention, the plasma concentration of the anti–IL-10 mAb (μg/mL; 2 μg/mL limit of detection indicated by horizontal dashed line; *n =* 6) (**C**) and the levels of plasma IL-10 (pg/mL; 10 pg/mL limit of detection indicated by horizontal dashed line; *n =* 6) (**D**) were measured by electrochemiluminescent sandwich immunoassay on a MesoScale platform. Plasma was drawn immediately before and 5 minutes after infusion of the anti–IL-10 mAb (d211 and d238 p.i.). (**B**–**D**) Individual RMs are given by color-coded, solid lines, and no statistics were performed. (**B**–**D**) ART is represented as a gray-shaded background, whereas anti–IL-10 mAb infusions are given by vertical dashed lines (purple) with the intervention phase amid ongoing ART represented as a purple-shaded background. (**E**) RNA-Seq was performed on PBMCs (*n =* 5) from the on-ART, treatment baselines (gray; d167 and d209 p.i.), and following administration of the anti–IL-10 mAb (purple; d229 and d263; as color-coded above). On individual RMs (as indicated above), sample-level enrichment analysis (SLEA) was performed to measure the expression of transcriptomic pathways associated with cell survival and differentiation (as indicated at left), and the enrichment score is given as a bidirectional heatmap (*P <* 0.05 for all shown measures).

To establish a causative link between levels of IL-10 and the maintenance of cell subsets critical for viral persistence in lymphoid tissues, a pilot study was performed to determine the impact of in vivo IL-10 neutralization during short-term ART. Specifically, 6 RMs were infected i.v. with SIVmac_239_ and at d35 p.i. began a daily ART regimen (dolutegravir, emtricitabine, tenofovir; Figure 5A and Supplemental Table 6). At d211 p.i. all RMs were i.v. administered 10 mg/kg of a rhesus-recombinant anti–IL-10 mAb (MK-1966/JES3.12G8). Notably, 2 RMs (RBf16 and RSr15) exhibited a slower control of plasma viremia while on ART, with RBf16 being detectable at the intervention baseline (40 copies/mL at d209 p.i.; Figure 5B). Four weeks later (d238 p.i.) all RMs received a second infusion of the anti–IL-10 mAb, which was dose-escalated to 20 mg/kg. The 2 doses were clinically well tolerated without reported adverse events and did not induce anti-drug antibodies as gauged by the stability of the plasma pharmacokinetics (Figure 5C). Neutralization resulted in a sustained, 2-log increase in levels of plasma IL-10, as determined by an electrochemiluminescent immunoassay (Figure 5D); although we cannot ascertain the ratio of mAb-bound versus unbound IL-10, these data are indicative of strong biological activity (i.e., inability of mAb-bound IL-10 to bind to IL-10R). Therefore, to ascertain whether in vivo neutralization was effective in inhibiting IL-10 signaling, RNA-Seq was performed on bulk PBMCs. By a sample-level enrichment analysis on individual RMs at discrete time points, IL-10 neutralization reduced the expression of genes associated with MYC pathways of cell survival, B cell differentiation, and Tfh cell homeostasis (Figure 5E).

### IL-10 neutralization impairs the survival of LN memory and CD4^+^ Tfh cells.

**Figure 6:**
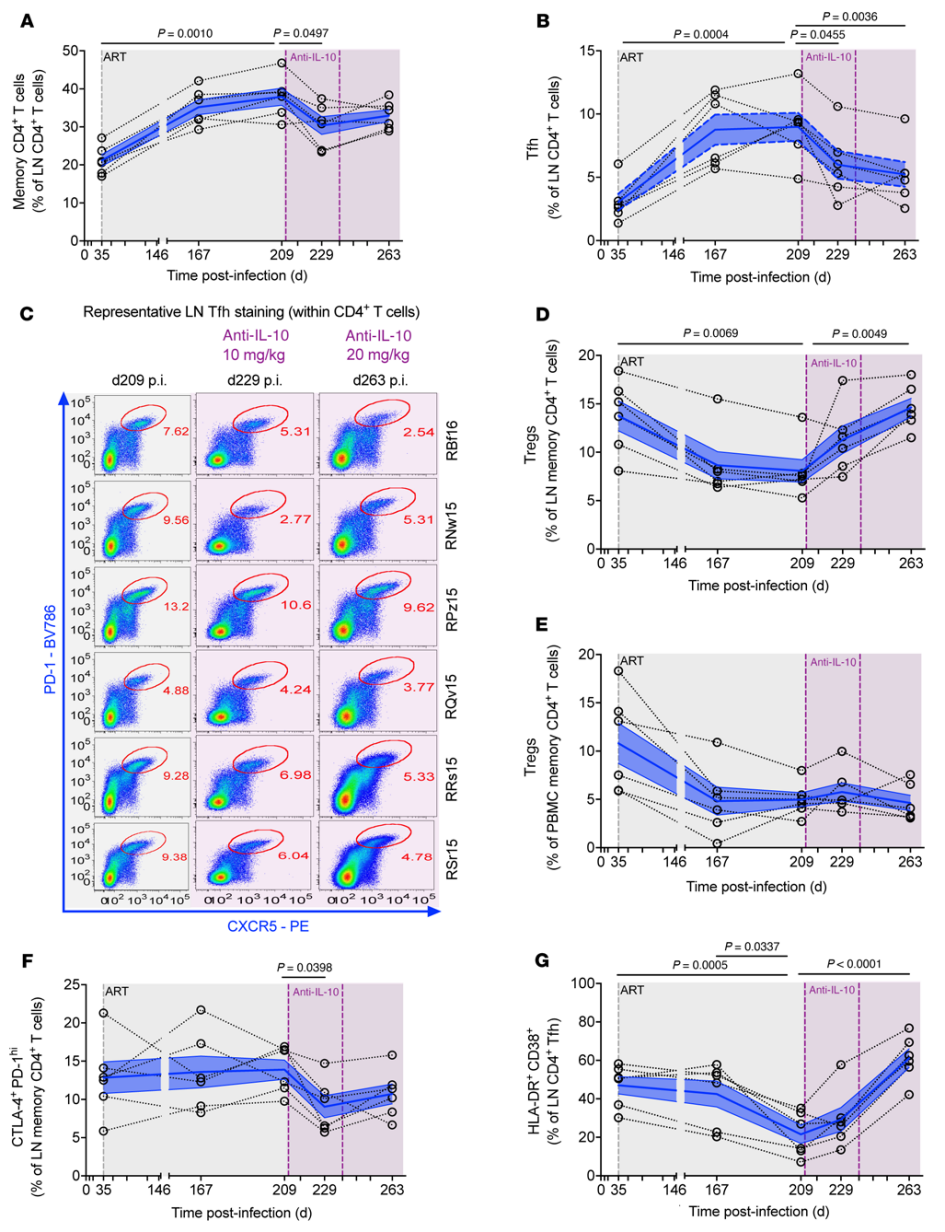
IL-10 neutralization impairs the survival of LN memory and CD4^+^ Tfh cells. (**A** and **B**) Flow cytometry was performed on fresh LN mononuclear cells (*n =* 6) in which the frequencies of total memory (CD95^+^) (**A**) and Tfh (CXCR5^+^PD-1^hi^) (**B**) cells among CD4^+^ T cells were quantified. (**C**) For the Tfh cell quantification, flow cytometry stains of CXCR5 against PD-1 inside the parental CD4^+^ T cell population are shown for all animals (*n =* 6, 18 of 30 unique; indicated at right) at the indicated time points (above). Gates and the percentage of parent are given in red. Time points analyzed include during chronic infection (d35 p.i.); the on-ART, pretreatment baselines (d167 and d209 p.i.); and at d18 (d229 p.i.) and d25 (d263 p.i.) after administration of 10 or 20 mg/kg of anti–IL-10 mAb, respectively. (**D**–**G**) Flow cytometry was also used to measure the frequency of Tregs (CD25^+^CD127^–^FoxP3^+^) among memory CD4^+^ T cells in LNs (**D**) and PBMCs (**E**); ICR coexpression, CTLA-4^+^PD-1^hi^, in memory CD4^+^ T cells (**F**); and activation (HLA-DR^+^CD38^+^) in CD4^+^ Tfh cells (*n =* 6 each) (**G**). (**A**, **B**, and **D**–**G**) Data from individual macaques are represented by tethered, open black circles with averaged data presented as mean (blue line) ± SEM (blue filled area) and were analyzed by 2-sided, 1-way ANOVA with matching using a Dunnett’s correction for multiple comparisons relative to the intervention baseline (d209 p.i.). ART is represented as a gray-shaded background, whereas anti–IL-10 mAb infusions are given by vertical dashed lines (purple) with the intervention phase amid ongoing ART represented as a purple-shaded background.

Supporting the hypothesis that IL-10–mediated p-STAT3 signaling favors cell survival and consistent with transcriptomic signatures in PBMCs upon IL-10 neutralization (Figure 5E), immunophenotyping in LNs showed a treatment-induced reduction in the frequency of memory CD4^+^ T cells and CD4^+^ Tfh cells (Figure 6, A–C; representative gating strategy shown in Supplemental Figure 4). Conversely, therapy increased the contribution of Tregs toward the pool of memory CD4^+^ T cells in LNs, but not in PBMCs (Figure 6, D and E). Notably, IL-10 neutralization also reduced the fraction of LN memory CD4^+^ and CD8^+^ T cells coexpressing CTLA-4 and PD-1 (Figure 6F and Supplemental Figure 5, A and B), suggesting either reversal of effector cell exhaustion leading to improved antiviral responses (55) and/or an elimination of CD4^+^ T cells that have been identified as enriched in replication-competent virus (4, 7). IL-10 neutralization also enhanced the frequency of immune activation (HLA-DR^+^CD38^+^) in LN memory CD4^+^ T cells, including within the Tfh cell (Figure 6G) and Treg subsets, and memory CD8^+^ T cells (Supplemental Figure 5, C–E), whereas enhanced cellular proliferation (Ki-67^+^) was limited to memory CD4^+^ T cells, including Tregs (Supplemental Figure 5, F–I). Collectively, these data demonstrate that targeting IL-10 signaling enables the reduction of cellular niches known to support viral persistence during ART.

### IL-10 neutralization reduces SIV-DNA content in LNs independent of SIV-specific T cell responses.

**Figure 7:**
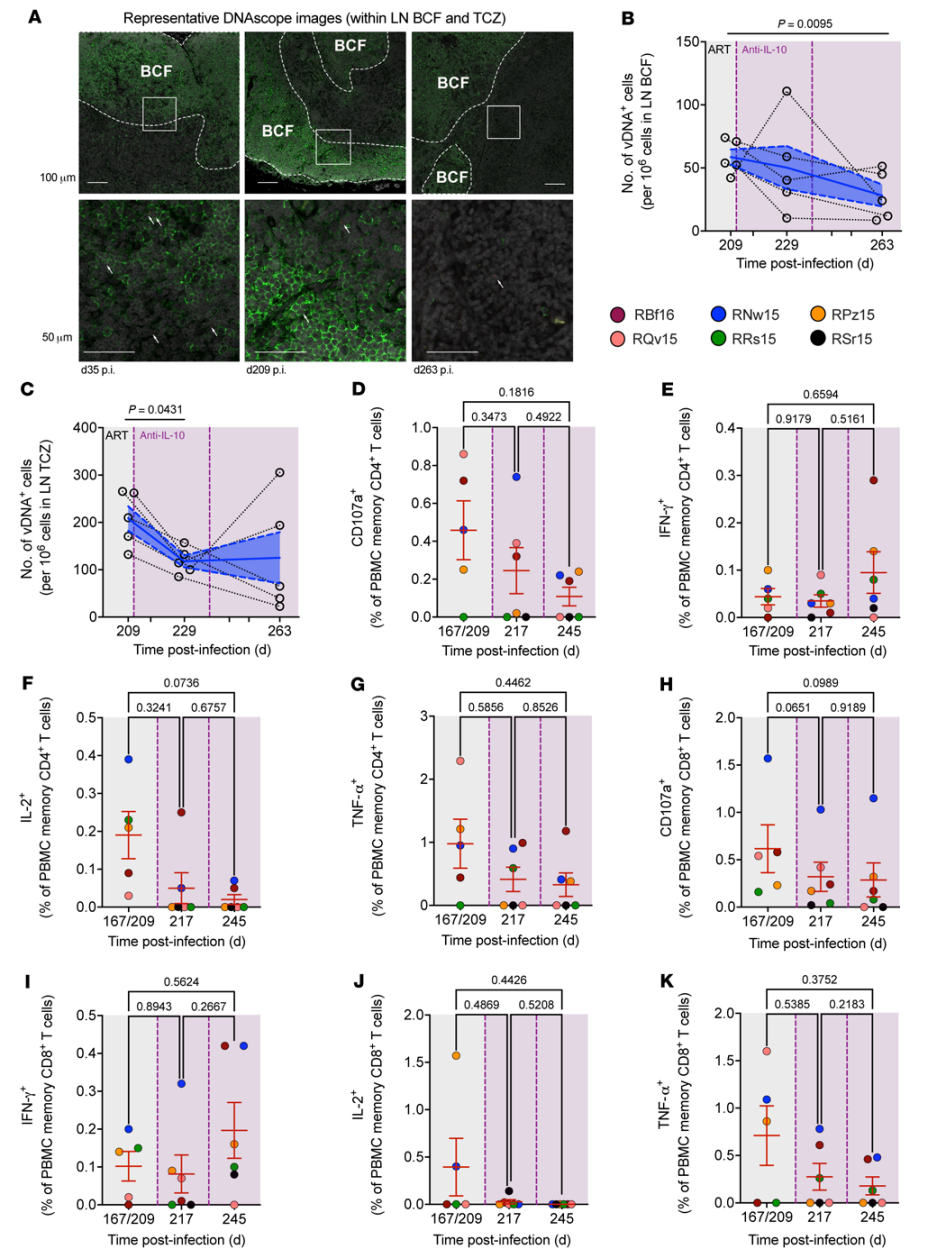
IL-10 neutralization reduces SIV-DNA content in LNs independent of SIV-specific T cell responses. (**A**) DNAscope in situ hybridization for cell-associated vDNA (red, indicated by arrows) was performed in combination with immunofluorescence imaging for a DAPI nuclear stain (gray) and CD20 (green) to define cell numbers and demarcate the BCF (white, dashed outlines), respectively. Representative DNAscope IHC and immunofluorescence images of formalin-fixed, paraffin-embedded LN from RRs15 at d35, d209, and 263 p.i. are shown (3 of 15 unique samples); bottom panels show boxed regions from top panels at higher magnification. Scale bars: 100, 50 μm. (**B** and **C**) Using these images, the vDNA content per 10^6^ cells was measured in the LN BCF (**B**) and TCZ (**C**) (*n =* 5 each). Data from individual macaques are represented by tethered, open black circles with averaged data presented as mean (blue line) ± SEM (blue filled area) and were analyzed by 2-sided, 1-way ANOVA with matching using Tukey’s correction for multiple comparisons between all factors. (**D**–**K**) Cryopreserved, rested PBMCs were stimulated with overlapping SIVmac_239_ GAG peptide pools, and by flow cytometry the frequency of CD107a^+^, IFN-γ^+^, IL-2^+^, and TNF-α^+^ cells was measured in memory CD4^+^ T cells (**D**–**G**) and memory CD8^+^ T cells (**H**–**K**), respectively (*n =* 6 each; RSr15 unavailable at baseline). Reported values have had background expression levels detected in the mock-stimulated condition subtracted. (**D**–**K**) Data from individual macaques are represented as color-coded circles with averaged data presented as mean ± SEM (red) and were analyzed by 2-sided, 1-way mixed-effects model using Tukey’s correction for multiple comparisons between all time points. (**D**–**K**) ART is represented as a gray-shaded background, whereas anti–IL-10 mAb infusions are given by vertical dashed lines (purple) with the intervention phase amid ongoing ART represented as a purple-shaded background.

Despite inducing proliferation (Ki-67^+^) in LN memory CD4^+^ T cells (Supplemental Figure 5F), IL-10 neutralization was insufficient to promote viral reactivation by plasma viremia (Figure 5B), the expression of p27 by Simoa in PBMCs or LN mononuclear cells (Supplemental Figure 6, A and B), or the levels of SIV-RNA^+^ cells in the LN BCF and TCZ as quantified by RNAscope (Supplemental Figure 6, C and D). Consistent with the reduction in the frequency of CD4^+^ T cell subsets known to contribute to viral persistence during ART (Figure 6, A–C), the levels of SIV-DNA^+^ cells, as measured by DNAscope in situ hybridization with immunofluorescence (representative image in Figure 7A), were reduced following IL-10 neutralization, in both TCZ and BCF (Figure 7, B and C). To investigate the mechanism by which the levels of SIV-DNA^+^ cells are limited, ex vivo stimulations were performed with PBMCs to gauge responsiveness of T cells to overlapping SIVmac_239_ GAG peptide pools. Despite earlier observations of reduced ICR expression (Figure 6F and Supplemental Figure 5, A and B), no enhancement was observed in cytokine (IFN-γ, IL-2, or TNF-α) or cytotoxic (CD107a) responses in memory CD4^+^ or memory CD8^+^ T cells at 1 week after treatment (Figure 7, D–K; representative gating strategy shown in Supplemental Figure 7), suggesting that reductions in viral burden are independent of increased SIV-specific T cell responses. Overall, despite being limited by the lack of a control arm, a small population size (*n* = 6), the short duration of ART, and only 2 administrations of the therapeutic, these data demonstrate that the rhesus-recombinant anti–IL-10 mAb can be administered in vivo and possesses prolonged plasma exposure. Furthermore, these observations validate that IL-10 signaling supports the maintenance and survival of memory CD4^+^ T cells, encompassing those expressing ICRs, such as Tfh cells, and those harboring SIV-DNA during ART.

## Discussion

One of the main barriers to designing and implementing therapeutic strategies to limit HIV persistence is the incomplete knowledge of mechanisms regulating the establishment and maintenance of the viral reservoir. Here, we have shown that plasma and LN levels of IL-10, as well as its transcriptomic signaling signatures, are induced by SIV infection to counterbalance proinflammatory responses, and remained elevated during ART. However, as IL-10 signaling regulates T cell differentiation and the survival of CD4^+^ T cell subsets infected by SIV, these physiologically elevated levels of plasma IL-10 contribute to seeding of the viral reservoir during chronic infection as evidenced by correlations with the content of cell-associated SIV-DNA in tissue, including within LN CD4^+^ Tfh cells. Furthermore, most follicular and non-follicular cells harboring cell-associated SIV-DNA were in close proximity to IL-10^+^ cells both during chronic infection and on ART, suggesting a role for IL-10 in supporting viral persistence. This observation is reinforced by data demonstrating that plasma levels of IL-10 and PBMC transcriptomic signatures of IL-10 signaling during chronic infection, including IL-10–regulated pathways of cell survival, predict the frequency of LN CD4^+^ Tfh cells during ART and their cell-associated SIV-DNA content, respectively. Altogether, our data indicate IL-10 signaling as a mechanism and correlate for the survival of CD4^+^ T cell subsets in which SIV/HIV may persist.

Our data show that a small increase in the physiological levels of plasma and lymphoid IL-10 that follows SIV infection produces meaningful alternations in transcriptomic signatures to promote T cell survival and differentiation. Furthermore, our IL-10 in vitro stimulations in uninfected PBMCs demonstrated a clear enhancement of p-STAT3, which is a critical regulator of Tfh cell differentiation and maintenance of the BCF (30–33). It is plausible that levels of IL-10 may have been higher either during acute infection (17) or following a more prolonged phase of chronic infection as a consequence of disease severity (56). Likewise, IL-10 levels may have fully normalized with a longer duration of ART, an inherent limitation of the nonhuman primate model. As longitudinal analyses were limited to elderly female macaques, we cannot rule out a role for sex or age in regulating levels of IL-10. As a caveat, we cannot rule out, and it is likely, that some vDNA^+^ cells do not express the IL-10R. Moreover, although we observed that cells harboring vDNA were often found in close proximity to IL-10–producing cells, we also observed a high degree of colocalization of uninfected cells with IL-10^+^ cells, and our data cannot support a role for IL-10 in preferentially supporting the survival of SIV-infected cells over uninfected cells. Likewise, as there is not an established link between productive SIV infection and the expression of IL-10R, is it unlikely that IL-10 supports the survival of cells harboring latent replication-competent virus relative to those with potentially defective or non-integrated (i.e., cell-associated) viral genomes. Thus, it is unlikely that IL-10 signaling is directly exploited by viral factors to promote the survival of a productively infected host cell, but rather it nonspecifically supports the homeostasis and long-term maintenance of a pool of target cells that contribute to viral persistence with long-term ART. If extrapolated beyond CD4^+^ T cells, our results on IL-10 signaling supporting the long-term maintenance of immunological memory may help explain recent data showing that IL-10R signaling favors the maintenance of a population of CD8^+^ T cells that promote tumor control in oncology models (57). Thus, our data suggest that IL-10 colocalization supports survival of T cells, including those harboring SIV-DNA, in immune-privileged anatomic niches of viral persistence during ART, such as the LN BCF (6, 58).

To demonstrate causality between IL-10 and the survival of CD4^+^ T cell subsets contributing to viral persistence, we performed a pilot study to evaluate the safety and bioactivity of IL-10 neutralization in SIV-infected RMs receiving short-term ART. Notably, this is the first time that IL-10 neutralization has been performed in SIV-infected RMs. The rhesus-recombinant anti–IL-10 mAb had stable plasma exposure across 2 administrations spaced by 4 weeks. Despite the short course of treatment, in LN memory CD4^+^ T cells, and specifically within LN CD4^+^ Tfh cells, IL-10 neutralization reduced cell survival in conjunction with limiting ICR coexpression, while increasing immune activation, thereby confirming that IL-10 regulates the maintenance of T cells that are key contributors to viral persistence during ART (4, 5, 7). As a caveat, we cannot rule out that residual IL-10 signaling occurred, as neutralization greatly expanded plasma levels of IL-10, which may have saturated out the therapeutic mAb. However, our transcriptomic data in PBMCs indicate that in vivo neutralization reduced signaling through IL-10–regulated pathways supporting Tfh cell and B cell homeostasis. Overall, these data demonstrate an encouraging potential to reduce the survival of cellular subsets preferentially infected by SIV by antagonizing IL-10 signaling without the need for viral reactivation.

These data raise the hypothesis that therapeutic strategies designed to inhibit IL-10 have the potential to impact the size of the HIV reservoir, particularly in LN follicles and CD4^+^ Tfh cells, key anatomic and cellular contributors to viral persistence during ART. Notably, studies have shown the ability of IL-10 to impair antiviral immune responses by inhibiting IFN-γ responses from HIV-specific CD4^+^ T cells, and inducing ICR expression on CD8^+^ T cells (16, 17, 23). Indeed, in mice, genetic deficiency of IL-10 and blockade of IL-10R have shown promise in promoting the clearance of chronic pathogens (22, 23). Thus, one can envision a scenario in which IL-10 blockade or neutralization may reduce the establishment and maintenance of the HIV reservoir by inhibiting pathways that favor CD4^+^ T cell survival and reducing the levels of CD8^+^ T cells expressing ICRs, which expression has been associated with dysfunctional antiviral immune responses (16, 23, 59–61). In conclusion, by promoting and predicting the maintenance of SIV-DNA^+^ cells, including CD4^+^ Tfh cells, IL-10 signaling contributes to SIV persistence during ART by promoting the survival of T cell subsets critical for viral persistence. Larger and longer controlled preclinical studies will be needed to evaluate whether IL-10 modulation, either alone or in combination with other immunotherapy approaches aimed at promoting latency reversal or CD8^+^ T cell responses, is safe and effective in promoting sustained SIV remission.

## Methods

### Macaque model for characterization of IL-10 levels and signaling.

Fifteen Indian-origin, specific pathogen–free (SPF) rhesus macaques (RMs; *Macaca mulatta*) were sourced from the Yerkes National Primate Research Center (YNPRC) colony and single-housed in an animal biosafety level 2 (BSL-2) facility at YNPRC as previously described (7). All RMs were female, between 54 and 120 months old at time of infection, and were *Mamu*-B*08^–^ and -B*17^–^ with 7 animals being *Mamu*-A*01^+^ (Supplemental Table 1). RMs were infected i.v. with 300 TCID_50_ (50% tissue culture infective dose) SIVmac_239_ and at day 60 post-infection (d60 p.i.) began a 5-drug, oral ART consisting of an integrase inhibitor (100 mg raltegravir/RLT twice a day), 2 nucleoside reverse transcriptase inhibitors (30 mg/kg emtricitabine/FTC and 20 mg/kg tenofovir/PMPA), and a boosted protease inhibitor (375 mg darunavir/DAR twice a day with 50 mg ritonavir/RIT). ART was maintained for 7 months with tissue biopsies collected at d259 p.i. Notably, RGe12 exhibited poor viral control. To increase the number of analyzed animals, our study include 7 RMs (RLm12, ROe12, RJp11, RGv10, RVt10, RTb12, RHa10) that, starting at d60 and d203 p.i., began a 6-dose, weekly cycle of subcutaneous rhesus IL-21-IgFc (IL-21) at 100 μg/kg (34), which did not impact plasma concentrations of IL-10, LN levels of IL-10, CD4^+^ T cell counts in peripheral blood, or the frequency of LN Tfh cells (Supplemental Figure 3, A and B, and ref. 34). Treatment group stratification was balanced for the set point viral loads and the nadir of CD4^+^ T cell counts during chronic infection.

### Macaque model for IL-10 neutralization study.

Six Indian-origin, SPF RMs (*M. mulatta*) were sourced from the YNPRC colony and single-housed at YNPRC in an animal BSL-2 facility as previously described (7). All RMs were male, between 43 and 46 months old at time of infection, and were *Mamu*-B*08^–^ and -B*17^–^ with 2 being *Mamu*-A*01^+^ (Supplemental Table 6). RMs were infected i.v. with 300 TCID_50_ SIVmac_239_ and at d35 p.i. began a 3-drug, daily ART regimen consisting of an integrase inhibitor (2.5 mg/kg dolutegravir/DTG; ViiV Healthcare) and 2 nucleoside reverse transcriptase inhibitors (40 mg/kg emtricitabine/FTC and 5.1 mg/kg tenofovir disoproxil fumarate/TDF; Gilead Sciences) coformulated in 15% kleptose (Roquette America) for subcutaneous administration (63). The anti–IL-10 mAb (see *Formulation of the anti–IL-10 mAb*) was delivered i.v. without pretreatment at d211 p.i. at 10 mg/kg and again 27 days later (d238 p.i.) at 20 mg/kg. Notably, RBf16 only received 14.45 mg/kg at the second anti–IL-10 mAb administration because of limited compound yields. Necropsy was performed on all RMs at d263 p.i. with tissues, including LN biopsies, collected postmortem. A schematic of the study design, generated with BioRender (biorender.com), is given in Figure 5A.

### Sample collection and processing.

LN biopsies, rectal biopsies, plasma, and PBMCs were collected longitudinally and processed as previously described (7, 34). In the IL-10 neutralization cohort, peripheral blood was drawn immediately before and 5 minutes after infusion of the anti–IL-10 mAb for pharmacokinetics and levels of plasma IL-10 (Figure 5, C and D).

### Formulation of the anti–IL-10 mAb.

The amino acid sequence of IL-10 is more than 95% identical between humans, cynomolgus macaques, and RMs; furthermore, the epitope targeted by the anti–IL-10 mAb is identical between humans and RMs. The anti–IL-10 mAb (MK-1966/JES3.12G8) is a κ chain IgG1 with a rhesus engineered variable domain and a rhesus constant domain, to minimize the formation of anti-drug antibodies. Before use, mAbs were purified using size exclusion chromatography and reverse-phase HPLC, and were confirmed as endotoxin free (<0.03 EU/mg). The route, dose, and interval for the anti–IL-10 mAb were selected based on a dose-ranging pilot conducted by Merck & Co. ,Inc. in which 0.3 and 10 mg/kg were tested in uninfected cynomolgus macaques. Pilot data suggested a 10 mg/kg dose would be sufficient to capture more than 90% of the antigen; however, deviations in pharmacokinetics and dynamics led to the recommendation to increase the dose to 20 mg/kg. Historically, no adverse events were encountered with 4 weekly administrations at 25 mg/kg using a humanized mAb in cynomolgus macaques or following a single infusion of 10 mg/kg in humans with systemic lupus erythematosus. Bioactivity of the rhesusized mAb was confirmed by the in vitro neutralization of induced rhesus STAT3 phosphorylation using a STAT3 capture antibody (clone 232209, catalog MAB1799, R&D Systems), p-STAT3 detection antibody (clone Tyr705/D3A7, catalog 9145L, Cell Signaling Technology), and an anti-rabbit secondary antibody (HRP conjugate, catalog 31460, Thermo Fisher Scientific) using a U937 reporter cell line (catalog CRL-1593.2, ATCC) engineered with a p-STAT3–responsive luciferase reporter using a lentiviral vector (catalog CLS-6028L-8, QIAGEN).

### Flow cytometry.

Fresh mononuclear cells (10^6^ cells per test) were stained with anti-human mAbs, as detailed in Supplemental Methods, that we have shown to be cross-reactive in RMs (7, 34, 64, 65) and that have been validated in databases maintained by the Nonhuman Primate Reagent Resource. Chemokine mAbs were incubated at 37°C for 15 minutes, and the surface stain was performed at room temperature for 30 minutes. In the characterization cohort, samples underwent fixation/permeabilization with BD Cytofix/Cytoperm for 17 minutes, and intracellular stains were performed for 30 minutes. In the neutralization cohort, these samples underwent fixation/permeabilization with a FoxP3/Transcription Factor Staining Buffer Kit (Tonbo Biosciences) at 4°C for 45 minutes. A representative stain is given in Supplemental Figure 4. Acquisition of phenotypic data was performed on a minimum of 100,000 live CD3^+^ T cells on an LSRII (BD Biosciences) or an LSRFortessa (BD Biosciences) driven by BD FACSDiva software. Acquired data were analyzed using FlowJo software (version 9.9.6).

### Fluorescence-activated cell sorting.

Cryopreserved PBMC- and LN-derived mononuclear cells from chronically infected (d58 p.i.) and ART-treated (d259 p.i.) RMs were sorted for the following memory CD4^+^ T cells subsets: naive (Tn; PD-1^dim^CD200^–^CD95^–^CD28^+^CCR7^+^), effector memory (Tem; PD-1^dim^CD200^–^CD95^+^CCR7^–^), central memory (Tcm; PD-1^dim^CD200^–^CD95^+^CCR7^+^), and T follicular helper (Tfh; CD200^+^PD-1^hi^). Samples were stained with a 9-parameter mAb panel: anti-CD3–APC-Cy7 (clone SP34-2; 5 μL; catalog 557757), anti-CCR7–Cy7PE (clone 3D12; 7.5 μL; catalog 557648), anti-CD28–PE-CF594 (clone CD28.2; 5 μL; catalog 562296), and anti-CD95–Cy5PE (clone DX2; 10 μL; catalog 559773), all from BD Biosciences; anti-CD200–PE (clone OX104; 10 μL; catalog 329206), anti–PD-1–BV421 (clone EH12.2H7; 5 μL; catalog 329920), and anti-CD4–BV650 (clone OKT4; 2.5 μL; catalog 317436), all from BioLegend; and anti-CD8–FITC (clone 3B5; 5 μL; catalog 50-113-7496) and LIVE/DEAD Fixable Aqua Dead Cell Stain (2 μL of 1:10 dilution; catalog L34957), both from Thermo Fisher Scientific. mAb volumes were scaled per 10^7^ mononuclear cells. Chemokine mAbs (e.g., CCR7) were incubated at 37°C for 15 minutes, and the surface stain was performed at room temperature for 30 minutes. Cells were sorted using a FACSAria II Flow Cytometer (BD Biosciences) in BSL-2^+^ containment driven with the BD FACSDiva software. A representative stain and sorting strategy is shown in Figure 2E.

### Quantitative viral outgrowth assay.

QVOA was performed as previously described (34, 66) on LN CD4^+^ cells. A detailed procedure is provided in Supplemental Methods.

### Soluble inflammation biomarkers.

Levels of soluble CD14 (sCD14), soluble CD163 (sCD163), neopterin, LPS, soluble IFN-γ–induced protein 10 (IP-10), and C-reactive protein (CRP) were quantified in plasma using commercially available ELISA kits according to the manufacturer’s instructions. sCD14 levels were quantified using a human CD14 Quantikine ELISA kit (R&D Systems) and expressed as μg/mL. IP-10 levels were quantified using a human IP-10 Quantikine ELISA kit (R&D Systems) and expressed as pg/ml. Plasma CRP levels were measured using a monkey CRP ELISA kit (Life Diagnostics Inc.) and expressed as μg/ml. sCD163 levels were quantified using a Macro163 ELISA kit (IQ Products and Trillium Diagnostics) and expressed as ng/mL. Neopterin levels were quantified using an ELISA for the quantitative determination of neopterin in serum, plasma, and urine (Brahms Diagnostica) and expressed as ng/mL. For quantification of LPS levels, plasma samples were diluted to 20% with endotoxin-free water and heated to 70°C for 10 minutes followed by quantification using a Limulus Amebocyte assay (Cambrex) expressed as pg/mL.

### Ultrasensitive soluble IL-10 immunoassay.

In the IL-10 characterization cohort, plasma IL-10 was determined by an ultrasensitive sandwich immunoassay developed at Merck & Co., Inc. on the Singulex Erenna Platform (EMD Millipore). Merck generated in-house mouse mAbs against human IL-10 (capture, TC50.31D11; detection, TC40.11D8), labeled by Singulex capture or detection antibody-labeling kits, as appropriate. The biotinylated capture antibody was immobilized on streptavidin-coated paramagnetic beads, which were incubated first with minimally diluted sample followed by fluorophore-labeled detection antibody. The detection antibody was then eluted from the bead, neutralized, and transferred to a 384-well plate. Mean fluorescent signal for each sample was directly proportional to the concentration of eluted antibody, which was itself proportional to the amount of bound analyte on the bead. The assay standard was recombinant nonhuman primate IL-10, expressed in Expi239F cells (catalog A14527, Thermo Fisher Scientific) and purified from supernatant. The assay was shown to be sensitive to 0.2 pg/mL of rhesus IL-10.

### Electrochemiluminescent IL-10 immunoassay.

In the IL-10 neutralization cohort, plasma IL-10 was measured with a less sensitive (10 pg/mL limit of detection) sequential-format electrochemiluminescent (ECL) sandwich immunoassay on a MesoScale Discovery Platform. The capture antibody, a biotinylated Merck mouse anti–human IL-10 mAb, was coated onto a streptavidin-coated assay plate. IL-10 from the sample was captured by the capture antibody. Assay standards were made by dilution of Merck rhesus-recombinant IL-10 in assay diluent containing 25 μg/mL rhesusized anti–IL-10 mAb (MK-1966/JES3.12G8) that was incubated for 1 hour before use. The detection antibody, a Merck in-house mouse anti–human IL-10 mAb modified by conjugation with Sulfo-Tag, was added to the assay plate. The assay plate was then read on the MesoScale Sector Imager, and the light signal generated was directly proportional to the concentration of IL-10 in the original sample.

### Pharmacokinetics of the anti–IL-10 mAb.

Detection of the anti–IL-10 mAb (MK-1966/JES3.12G8) was performed by an ECL sandwich immunoassay in homogeneous format on a MesoScale Discovery Platform with a 2 μg/mL limit of detection. Capture and detection antibodies were Merck in-house mouse mAbs against the drug antibody idiotype. The capture antibody was biotinylated, and the detection antibody was conjugated to Sulfo-Tag. The assay buffer contained 20% rhesus serum, to ensure a constant serum level in all samples, calibrators, and controls regardless of dilution. The capture antibody, detection antibody, and sample were mixed and incubated in a nonbinding plate to allow complex formation. The incubated mixture was then added to a streptavidin-coated assay plate and incubated to allow complex formation. The plate was then read on a MesoScale Sector Imager, with the light signal generated by each sample being directly proportional to the drug concentration in that sample.

### Intracellular p27 single-molecule array assay.

Cryopreserved PBMCs and LN mononuclear cells were thawed and lysed at 5 × 10^6^ cells/mL with lysis buffer containing 1% Triton X-100 in 50% FBS and 0.5% casein in PBS (67). The lysates were frozen at –80°C overnight and were then incubated with 50 μL Dynabeads M-280 Streptavidin (catalog 11206D, Thermo Fisher Scientific) for 3 hours with rotation using a HulaMixer. The lysate was centrifuged at 18,407*g* for 10 minutes, and the supernatant was collected. For the Single Molecule Array Assay (Simoa), a Quanterix p24 kit was used with standard assay conditions with the substitution of an anti-p27 detection mAb (catalog ABL-4324, ABL Inc.), which was biotin-labeled and used at a final working concentration of 0.3 μg/mL. The p27 concentration (pg/mL) was calculated based on the assay standard curve.

### Viral loads.

Plasma SIV viral loads (SIV-RNA copies/mL) were determined using quantitative reverse transcription PCR (RT-qPCR) assays with either a 60-copies/mL (68) or a 15-copies/mL (69) limit of detection for the IL-10 characterization and neutralization cohorts, respectively.

### Cell-associated SIV-DNA and -RNA.

In the IL-10 characterization cohort, cryopreserved, sorted memory CD4^+^ T cell subsets from PBMCs and LN biopsies were analyzed for cell-associated SIV-DNA content using a modified version of a quantitative, nested PCR assay as previously described (34, 70).

### IL-10 in vitro stimulations.

Viable, frozen RM PBMCs from healthy donors were thawed, counted, and rested for 2 hours at 37°C, 5% CO_2_, at a concentration of 2 × 10^6^ PBMCs per milliliter in vented cap bottles with RPMI 1640 (MilliporeSigma) supplemented with 5 mM 4-(2-hydroxyethyl)-1-piperazine-ethanesulfonic acid (HEPES; Corning), 2 mM glutamine (UCSF Cell Culture Facility), 50 μg/mL penicillin/streptomycin (Corning), 5 mM sodium pyruvate (Corning), and 10% FBS (Gibco). Upon resting, 1 × 10^6^ PBMCs were transferred to a 48-well plate and left unstimulated or stimulated with IL-10 (5 ng/mL; catalog 200-10, PeproTech) with or without anti–IL-10 (MK-1966/JES3.12G8, Merck). To evaluate IL-10 signaling through phosphorylation of STAT3 (p-STAT3; clone 4/P-STAT3, BD Biosciences), different doses of anti–IL-10 were used (0.01–100 μg/mL). Frequencies of p-STAT3^+^ cells were evaluated after 30 minutes of stimulation by flow cytometry. The expression of Bcl-6 (a Tfh cell transcription factor; clone K112-91, BD Biosciences) or ICR markers (PD-1, clone EH12.2H7, BioLegend; and CTLA-4, clone BNI3, BD Biosciences) was evaluated by flow cytometry after 48-hour stimulation.

### RNA-Seq collection and analysis.

PBMCs were stored in RLT buffer (QIAGEN) at –80°C and later extracted using RNeasy kits (QIAGEN). RNA-Seq data were collected at the Yerkes Nonhuman Primate Genomics Core laboratory as previously described (34). Gene set enrichment analysis (GSEA) was performed using a compiled set of pathways from public databases including MSigDB version 5.1 (http://software.broadinstitute.org/gsea/msigdb/) and blood cell marker signatures (62). To test for the enrichment of Tfh cell differentiation (Figure 5E), we used an in-house signature (Malika Aid, unpublished observations) for which component genes are listed in the source data (see *Data and code availability*). The GSEA Java desktop program was downloaded from the Broad Institute (http://www.broadinstitute.org/gsea/index.jsp) and used with GSEAPreranked module parameters (number of permutations: 1000; enrichment statistic: weighted; seed for permutation: 111; 10 ≤ gene set size ≤ 5000). We used the Dynet Analyzer application implemented in Cytoscape version 3.6.0 to generate gene interacting networks to highlight overlapping genes between the different enriched modules. Sample-level enrichment analysis (71) was used to investigate the enrichment of pathways in individual RMs upon IL-10 neutralization. Briefly, the expression of all the genes in a specific pathway was averaged across samples and compared with the average expression of 1000 randomly generated gene sets of the same size. The resulting *z* score was then used to reflect the overall perturbation of each pathway in each individual sample.​ Data were visualized using ggplot2 (version 3.3.2) in RStudio (version 1.4.1103) with custom code.

### Immunohistochemistry.

IL-10 IHC in formalin-fixed, paraffin-embedded (FFPE) LNs was essentially performed as previously described (72) and is described in detail in Supplemental Methods for mouse IL-10 (1–2 μg/mL; clone E-10, Santa Cruz Biotechnology).

### SIV-RNA chromagen in situ hybridization.

RNAscope was performed on formaldehyde-fixed, paraffin-embedded tissue sections (5 μm) according to our previously published protocol (28) with minor modifications detailed in Supplemental Methods.

### SIV DNAscope with multiplex immunofluorescence.

Next-generation DNAscope in situ hybridization and multiplex immunofluorescence staining were performed on FFPE LN biopsies (Supplemental Table 4) as previously described (28) with some modifications as detailed in Supplemental Methods.

### Peptide stimulations.

As previously described (49), cryopreserved PBMCs were thawed and rested overnight (37°C, 5% CO_2_) at a concentration of 2 × 10^6^ cells/mL in R10 media. For the ex vivo stimulations, 10^6^ cells were cultured for 6 hours (37°C, 5% CO_2_) in R10 media at 10^6^ cells/mL in the presence of anti-CD28–BUV737 mAb (clone CD28.2; 10 μg/mL; BD catalog 612815), anti-CD49d mAb (clone 9F10; 4 g/mL; BD catalog 340976), and anti-CD107a–BV711 mAb (clone H4A3; 5 μL; BD catalog 563869) in conjunction with either 0.5% vol/vol DMSO (i.e., “mock”), freshly reconstituted peptide pools (2 μg/mL per each peptide, i.e., “stimulated”), or Staphylococcal Enterotoxin B (2 μg/mL, i.e., “positive control”; List Biologicals catalog 122). Overlapping SIVmac_239_ GAG peptide pools (catalog ARP-12364) were obtained through the HIV Reagent Program, Division of AIDS, National Institute of Allergy and Infectious Diseases (NIAID), NIH. A Protein Transport Inhibitor Cocktail (1:500 dilution; eBioscience catalog 00-4980-03), which contains brefeldin A and monensin, was added to each condition at 2 hours after initiation. Samples were washed twice with PBS and immunophenotyped with the rhesus-reactive mAbs detailed in Supplemental Methods. A representative stain is given in Supplemental Figure 7. Surface and intracellular stains were incubated at room temperature for 30 minutes, and cells underwent fixation/permeabilization with a FoxP3/Transcription Factor Staining Buffer kit (Tonbo Biosciences catalog TNB-0607-KIT) at 4°C for 45 minutes. Acquisition of phenotypic data was performed on a minimum of 120,000 live CD3^+^ T cells on a FACSymphony A5 (BD Biosciences) driven by BD FACSDiva software. Acquired data were analyzed with FlowJo software (version 10.8.0). Shown values of IL-2, TNF-α, IFN-γ, and CD107a expression were calculated by subtraction of the mock-stimulated background from the peptide-stimulated condition per each sample with negative values being set to zero. Gates were set back on biomarker expression within the naive T cell subsets. The baseline measurement uses PBMCs from both d167 (RRs15, RNw15) and d209 (RQv15, RPz15, RBf16) owing to limited sample availability; RSr15 had no cells available for analysis.

### Material transfer agreements.

This study used a novel anti–IL-10 mAb (MK-1966/JES3.12G8) that is a proprietary reagent developed by Merck & Co., Inc. The anti–IL-10 mAb is subject to material transfer agreement restrictions as the therapeutic is currently under investigation in human clinical trials.

### Data and code availability.

Source data and custom code supporting this work are available from the corresponding author upon reasonable request. RNA sequencing data were previously deposited in the NCBI’s Gene Expression Omnibus (https://www.ncbi.nlm.nih.gov/geo/query/acc.cgi) and are publicly available under GEO accessions GSE196182 for the IL-10 characterization cohort and GSE196436 for the IL-10 neutralization cohort. GSEA was performed using a compiled set of pathways from public databases including MSigDB version 5.1 (http://software.broadinstitute.org/gsea/msigdb/) and those previously reported (62), or with in-house signatures (Malika Aid, unpublished observations) for which component genes are listed in the source data. RNA-Seq data were visualized with ggplot2 (version 3.3.2) in RStudio (version 1.4.1103) with custom code (https://github.com/JustinLeviHarper/IL-10-RNA-seq-Visualization).

Requests for materials should be sent to Bonnie J. Howell.

### Statistics.

Data sets were tested for a Gaussian distribution using the D’Agostino-Pearson omnibus normality test. Correlations against plasma IL-10 concentrations were conducted using either 2-sided Pearson’s or nonparametric Spearman’s analyses as indicated. Univariate comparisons between data at 2 time points were analyzed with either a 2-sided Mann-Whitney *U* test or a Wilcoxon’s matched-pairs signed rank test dependent on replicate matching. Univariate longitudinal and multivariate cross-sectional data sets were analyzed with a 2-sided, 1-way ANOVA, or mixed-effects model if data points were missing, using the indicated correction for multiple comparisons. All statistical tests were performed 2-sided with a 95% confidence interval and adjusted for multiple comparisons, where applicable. Data showing averaged statistical outcomes are represented as mean ± SEM, and population sizes are listed in the figure legends per each analysis. The above statistical analyses were performed using GraphPad Prism 9.0.1. The assessment of multicollinearity was determined by multiple linear regression analysis in SAS (version 9.4, SAS Institute Inc.; ref. 73). RT-qPCR data from CD4^+^ subsets with fewer than 10,000 sorted events were excluded from analyses as previously determined based on the assay’s limit of detection. RBf16 from the IL-10 neutralization cohort was excluded from DNAscope analyses owing to poor sample quality. RNA-Seq data were analyzed with GSEAPreranked module parameters (number of permutations: 1000; enrichment statistic: weighted; seed for permutation: 111; 10 ≤ gene set size ≤ 5000). Investigators were not blinded to treatment conditions or the relative experimental phases from which samples were acquired.

### Study approval.

This study was approved by the Emory University Institutional Animal Care and Use Committee via permits 2001973 and 2003576. Experiments were conducted following guidelines set forth by the NIH and the Animal Welfare Act in regard to the housing and welfare of laboratory animals. All possible efforts were made to minimize pain experienced by the animals.

## Author contributions

Conceptualization: BJH, SPR, DG, DJH, RPS, and M Paiardini conceptualized the study. JH, SPR, CNC, CD, LM, M Pino, BC, GR, ER, GA, GW, and RJOB carried out the investigations. JH, CNC, CD, MA, NS, SEB, and KE conducted formal analyses of the data. GQDP and KBS provided resources. JH and M Paiardini wrote the original draft of the manuscript. JH, SPR, KE, DG, JDE, RPS, and M Paiardini wrote, reviewed, and edited the manuscript. JH, CNC, and MA generated figures. DG, JDE, RPS, and M Paiardini supervised the study. GS, DAK, JDE, RPS, and M Paiardini acquired funding. The ordering of shared first authorship was determined by their relative contributions to the study: JH performed the in vivo nonhuman primate studies, analyzed the flow cytometry and viral reservoir data, and wrote the manuscript, whereas SPR performed the in vitro stimulations and contributed to conceptualization of the study.

## Supplementary Material

Supplemental data

Supplemental data set 1

## Version 1 03/01/2022

In-Press Preview

## Version 2 04/15/2022

Electronic publication
