# Supplementary material for: Interleukin-10 contributes to reservoir establishment and persistence in SIV-infected macaques treated with antiretroviral therapy
Source: J Clin Invest. 2022 Apr 15;132(8):e155251. doi: 10.1172/JCI155251 (PMC9012284; doi:10.1172/JCI155251)
Supplement: Supplemental data [file jci-132-155251-s008.pdf]

Figure S1. IL-10 in vitro stimulation induces expression of proteins with exhaustion, survival, and immune-modulatory functions.

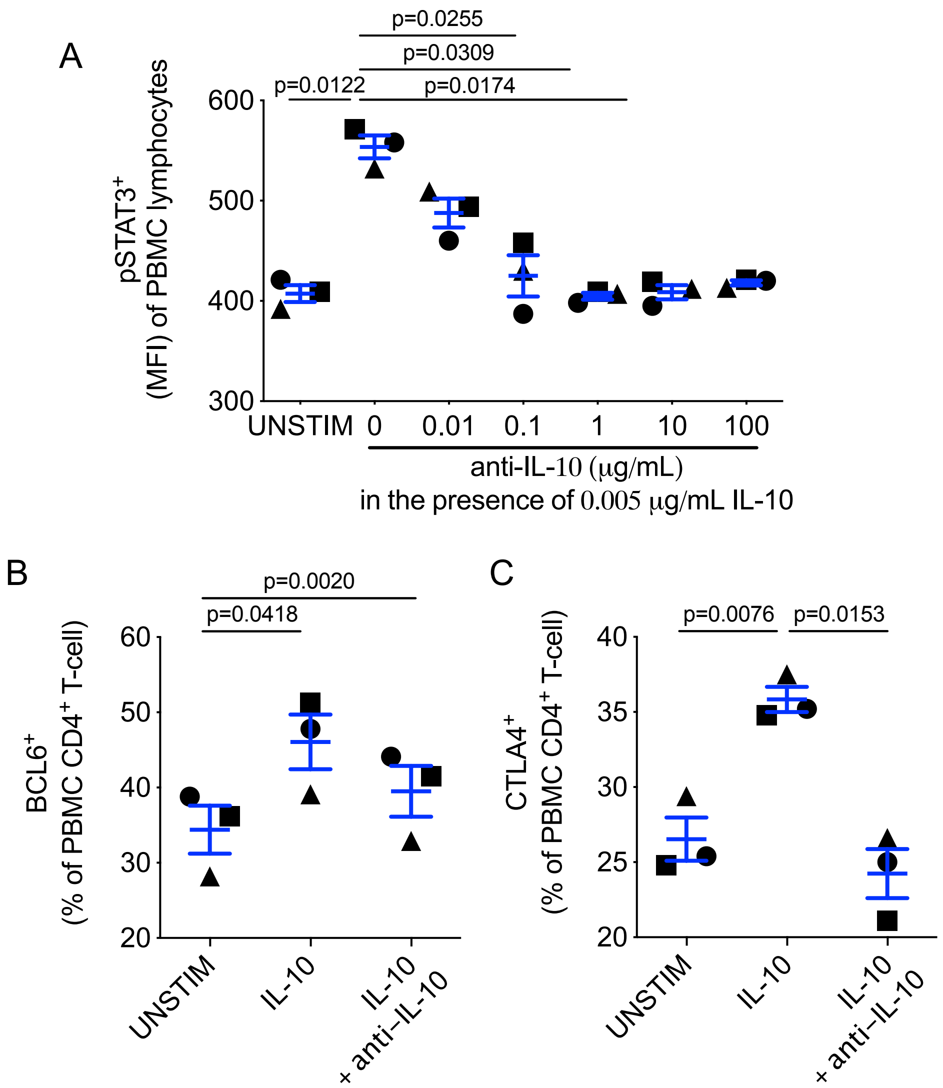

PBMCs from uninfected (SIV$^-$) rhesus macaques (RMs) were either unstimulated (UNSTIM) or stimulated with 0.005 μg/mL rhesus recombinant IL-10 (IL-10) alone or in the presence of an anti-IL-10 mAb in either (**A**) a 10-fold concentration gradient ranging from 0.01 to 100 μg/mL, or (**B**,**C**) at 10 μg/mL. Flow cytometry was utilized to quantify the (A) mean fluorescence intensity (MFI) of pSTAT3 in lymphocytes, and the frequency of (B) BCL-6$^+$ and (C) CTLA-4$^+$ CD4$^+$ T-cells (n=3 each). (A,B,C) Data from individual RMs is represented as black, filled symbols with averaged data presented as the mean ± SEM (blue). Data was analyzed using a two-sided, one-way repeated measures ANOVA with Tukey correction for multiple comparisons against all conditions.

Figure S2. In chronically infected RMs, plasma IL-10 correlates with $CD4^+$ T-cell depletion and inflammation.

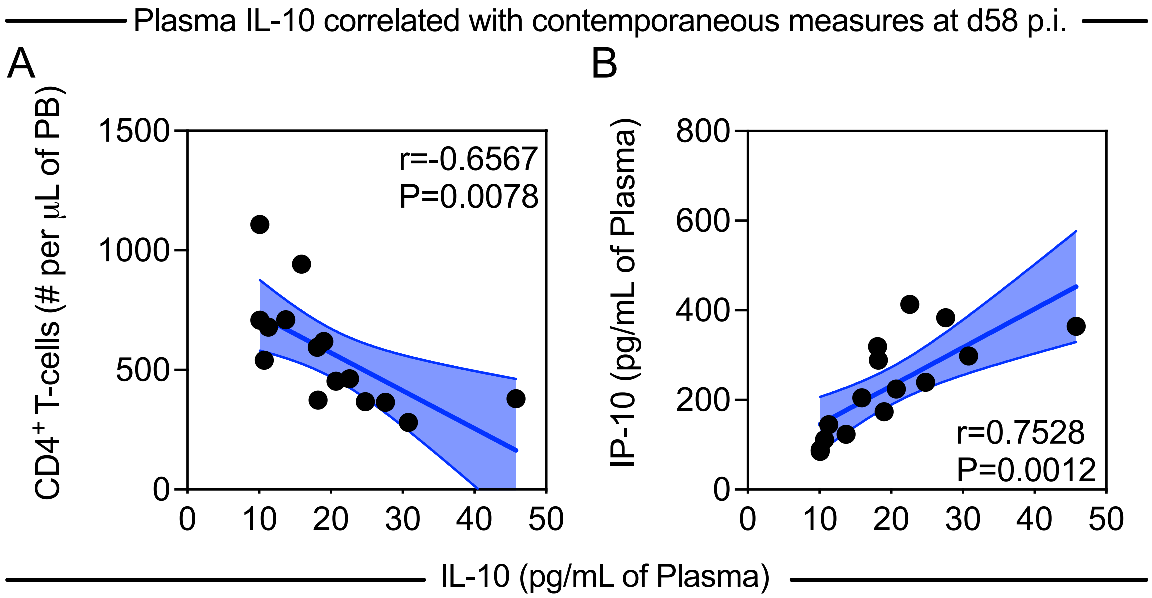

Plasma concentrations of IL-10 (pg/mL) during chronic infection (d58 post-infection; p.i.) were correlated against (**A**) the absolute number of $CD4^+$ T-cells per μL of peripheral blood (PB) by flow cytometry and (**B**) levels of plasma soluble IFN-γ-induced protein 10 (IP-10; pg/mL) by ELISA at d58 p.i. (n=14 each). (A,B) Data from individual macaques are represented as black circles and are overlaid with a simple linear regression (solid blue line) with a 95% confidence interval (blue fill). Correlations were performed using a two-sided Pearson correlation coefficient.

Figure S3. Plasma IL-10 concentrations and the frequency of LN CD4⁺ T_{FH} were unaffected by prior IL-21 treatment at ART initiation.

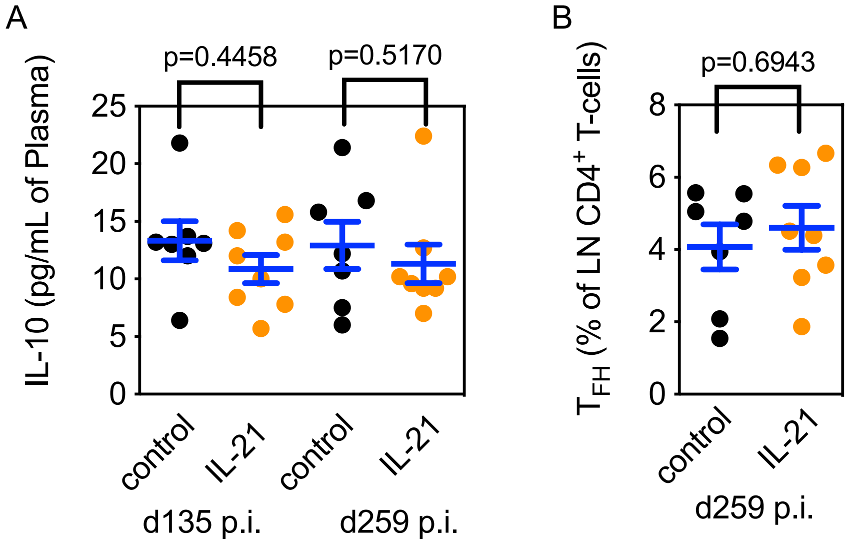

Parameters used for IL-10 correlations were stratified by prior treatment status: (**A**) the concentration of plasma IL-10 (pg/mL) by ultrasensitive sandwich immunoassay and (**B**) the frequency of LN $CD4^+$ T follicular helper cells (T_{FH}; $CXCR5^+PD\text{-}1^{hi}$) by flow cytometry. (A,B) Antiretroviral therapy (ART)-treated macaques are separated by treatment status: rIL-21-IgFc (n=8, orange) or control (n=7, black). Averaged data are presented as the mean ± SEM (blue). Statistics were performed with a two-sided Mann-Whitney U test.

Figure S4. Representative flow cytometry gating strategy used for longitudinal immunophenotyping of the IL-10 neutralization cohort.

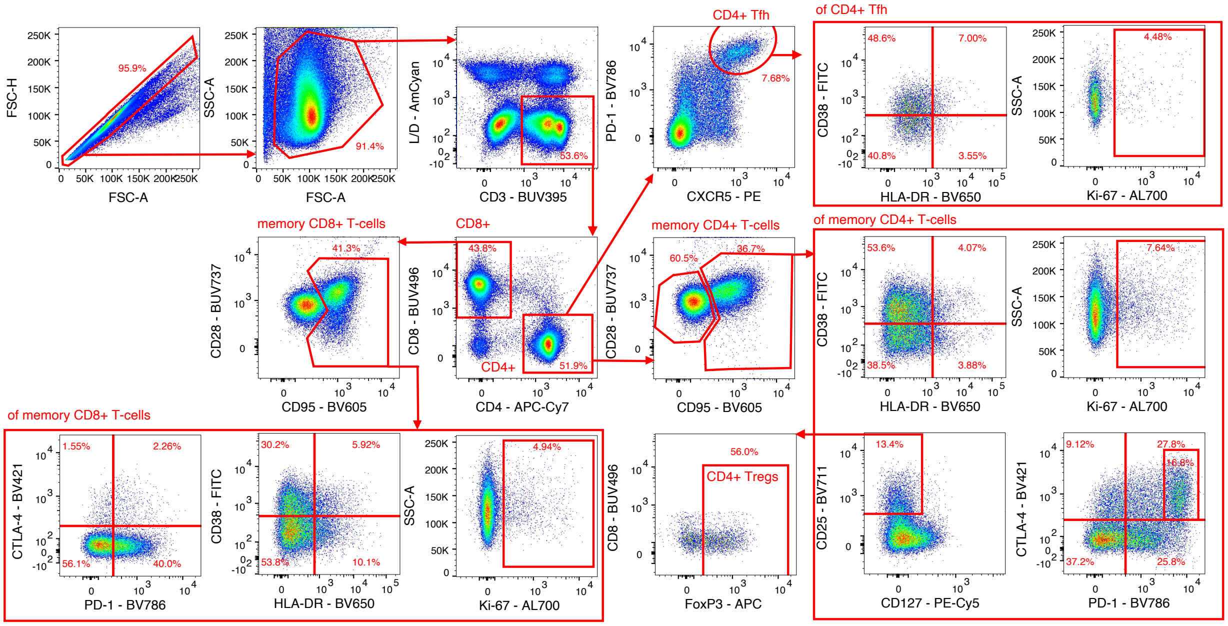

The flow cytometry gating strategy used for the immunophenotyping of fresh mononuclear cells in the IL-10 neutralization cohort is represented as a series of pseudo color plots. Gates are shown in red with arrows indicating the gating hierarchy. Clusters of biomarkers within a common parental population are outlined by a red box. T-cell subsets of interest are indicated in red, overlaid text. Shown are lymph node (LN) mononuclear cells from RBf16 at the pre-intervention baseline (d209 post-infection.; 1 of 30 unique LN samples) collected on an LSRFortessa (BD Biosciences).

Figure S5. IL-10 neutralization enhances T-cell activation and reduces ICR expression.

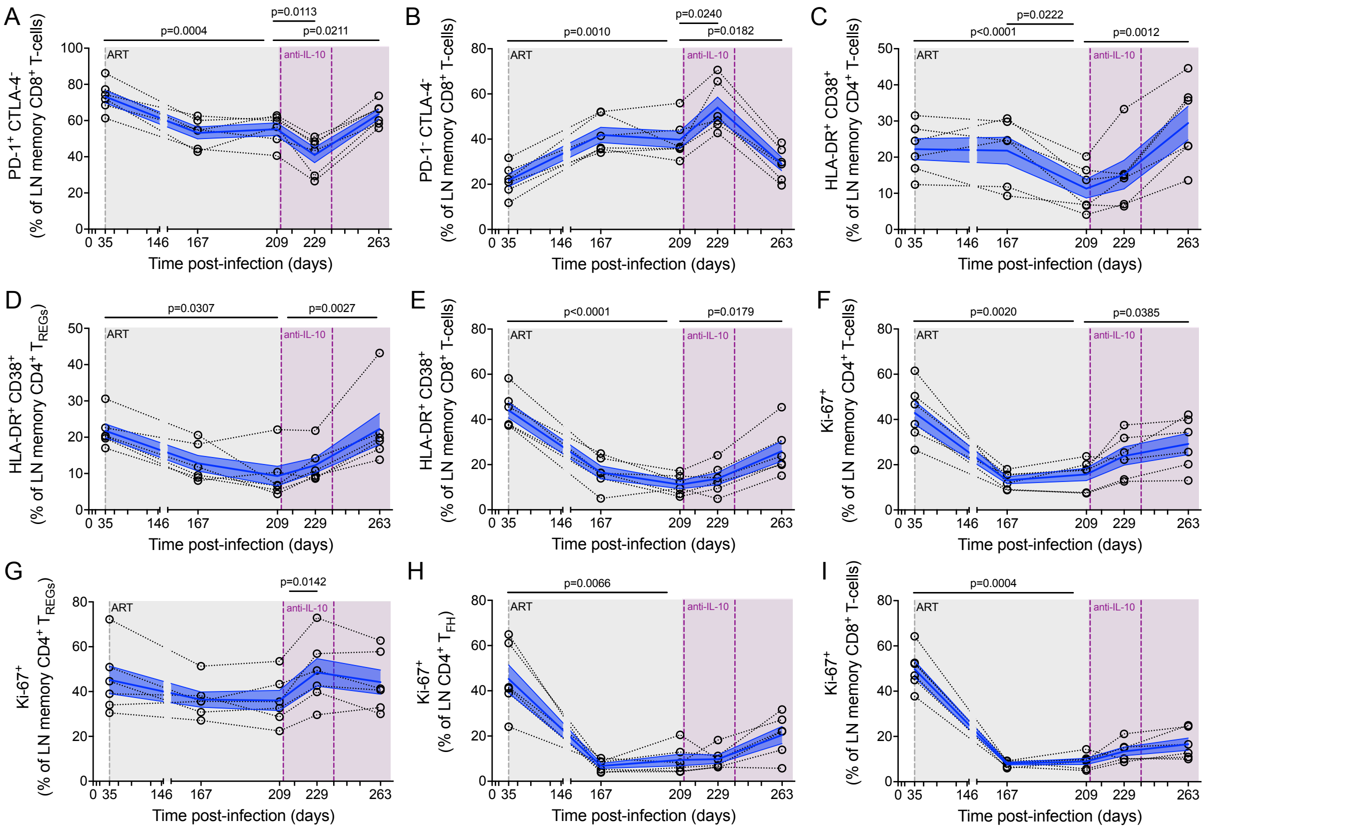

Flow cytometry was used to quantify the expression of immune checkpoint receptors as a frequency of memory ($CD95^+$) $CD8^+$ T-cells in lymph node (LN) mononuclear cells: (**A**) %PD-1^+CTLA-4^- and (**B**) %PD-1^-CTLA-4^-. Immune activation (HLA-DR^+CD38^+) was quantified in (**C**) memory $CD4^+$ T-cells, (**D**) memory $CD4^+$ T regulatory cells (T_{REGs}; $CD25^+CD127^-FoxP3^+$), and (**E**) memory $CD8^+$ T-cells. Cellular proliferation (Ki-67^+) was measured in (**F**) memory $CD4^+$ T-cells, (**G**) memory $CD4^+$ T_{REGs}, (**H**) $CD4^+$ T follicular helper (T_{FH}) cells and (**I**) memory $CD8^+$ T-cells (n=6 each). Data from individual macaques are represented by tethered, open black circles with averaged data presented as the mean (blue line) ± SEM (blue filled area) and was analyzed with a two-sided, one-way ANOVA with matching using a Dunnet correction for multiple comparisons relative to the intervention baseline (d209 p.i.). Antiretroviral therapy (ART) is represented as a grey shaded background; whereas, anti-IL-10 mAb infusions are given by vertical dashed lines (purple) with the intervention phase amid ongoing ART represented as a purple shaded background.

Figure S6. IL-10 neutralization during ART does not induce viral reactivation in lymphoid tissue.

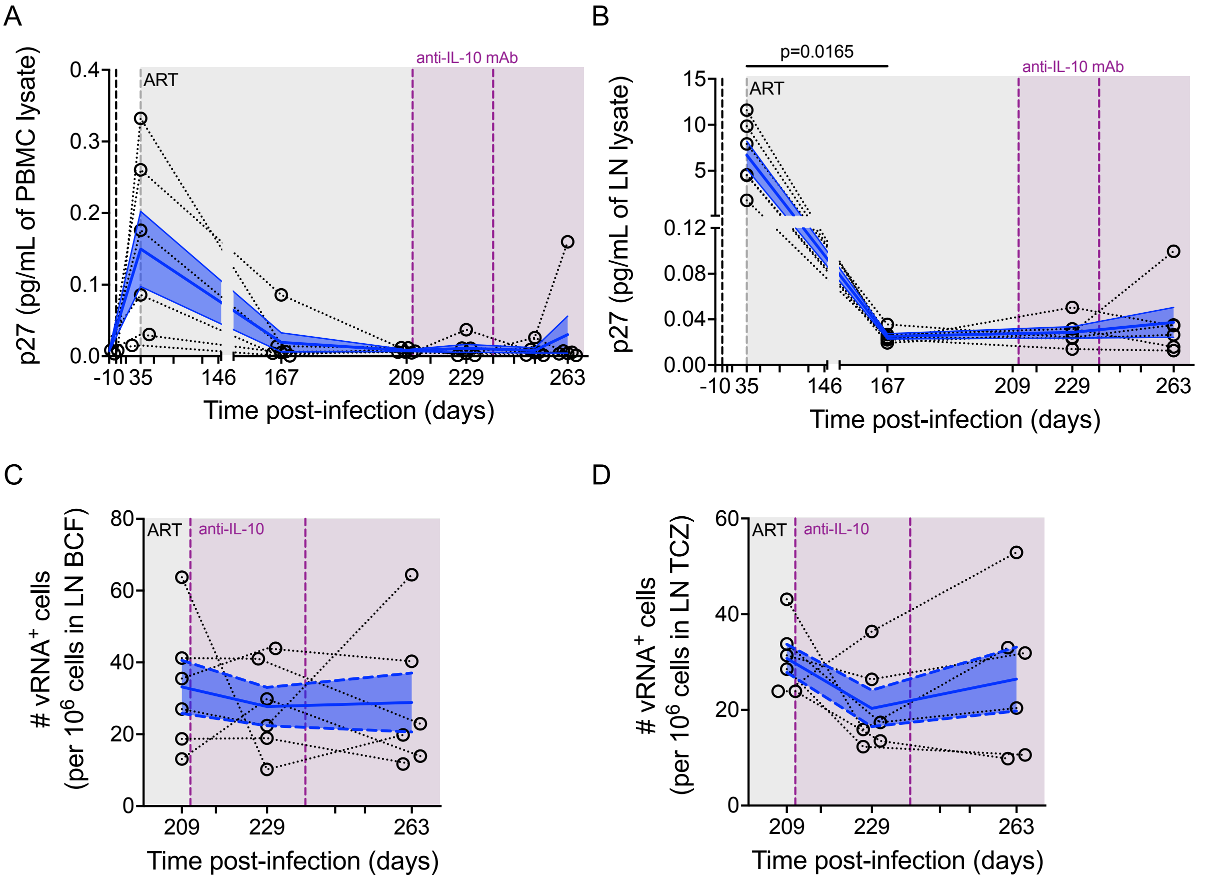

SIV p27 (pg/mL) was longitudinally measured by Single Molecule Array (Simoa) in the lysate of (**A**) PBMCs and (**B**) lymph node (LN) mononuclear cells (n=6 each). On formalin-fixed, paraffin-embedded LN (n=6), RNAscope *in situ* hybridization for cell-associated viral RNA (vRNA) was performed in combination with immunofluorescence imaging for DAPI nuclear stain and CD20 to quantify the vRNA content per 10^6 cells in the (**C**) B-cell follicle (BCF) and (**D**) T-cell zone (TCZ). (A,B,C,D) Data from individual macaques is represented by tethered, open black circles with averaged data presented as the mean (blue line) ± SEM (blue filled area). Antiretroviral therapy (ART) is represented as a grey shaded background; whereas, anti-IL-10 mAb infusions are given by vertical dashed lines (purple) with the intervention phase amid ongoing ART represented as a purple shaded background. Data was analyzed with either a (A,B) two-sided, one-way ANOVA with matching using a Dunnet correction for multiple comparisons relative to the intervention baseline or (C,D) a two-sided, one-way ANOVA with matching using a Tukey correction for multiple comparisons between all time points.

Figure S7. Representative flow cytometry gating strategy used for ex vivo peptide stimulations.

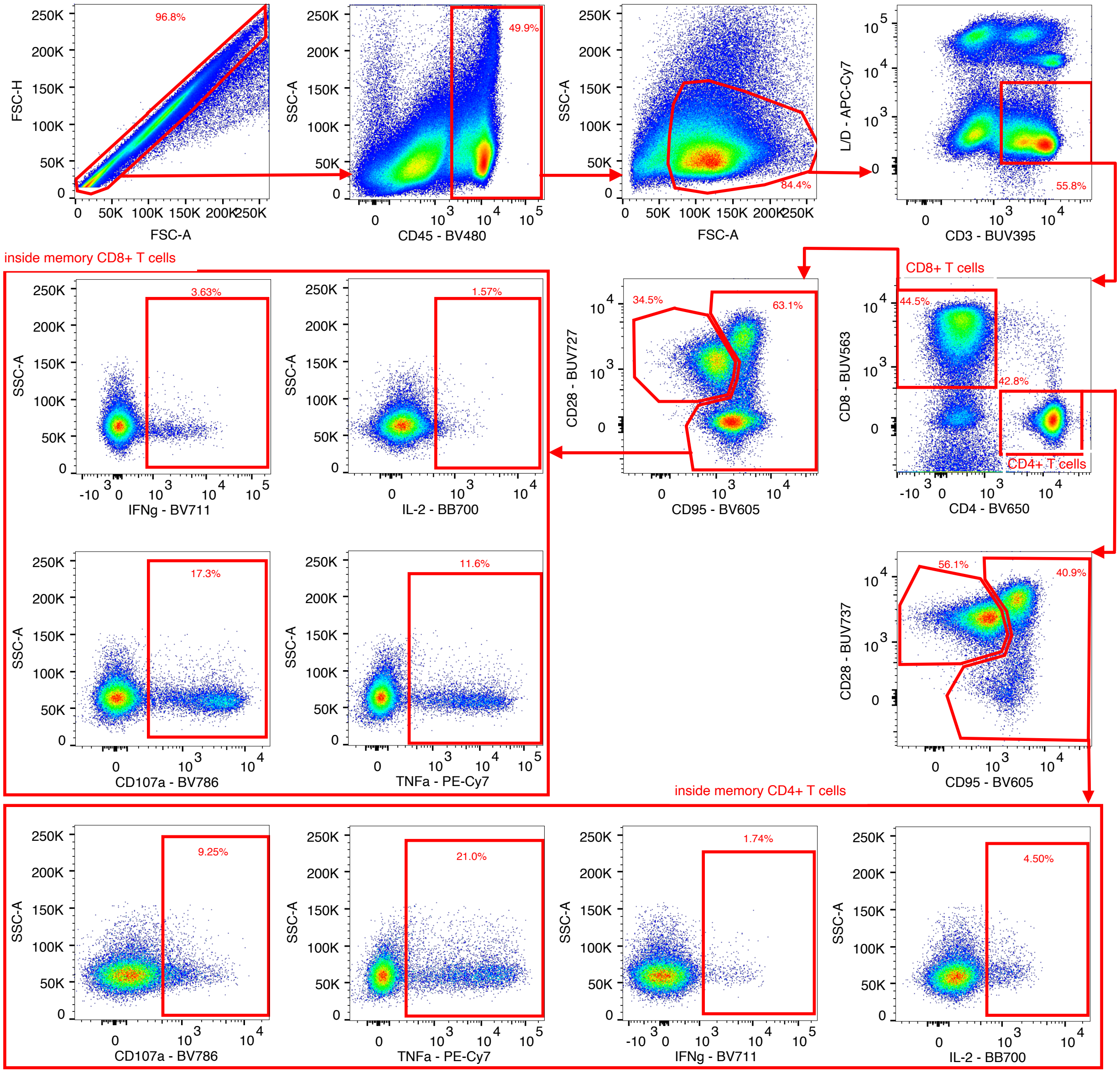

The flow cytometry gating strategy used for the immunophenotyping of cytokine and degranulation response of cryo-preserved PBMCs to ex vivo SIV-GAG peptide stimulation is represented as a series of pseudo color plots. Gates and the percentage of a population within the parental are shown in red with arrows indicating the gating hierarchy. Clusters of biomarkers within a common parental population are outlined by a red box. T-cell subsets of interest are indicated in red, overlaid text. Shown are Staphylococcal Enterotoxin B (SEB)-stimulated PBMCs (i.e. positive control) from RBf16 at the pre-intervention baseline (d209 post-infection; 1 of 3 unique SEB-stimulated samples) collected on an FACSymphony A5 (BD Biosciences).

Table S1. Characteristics of rhesus macaques utilized for longitudinal phenotypic and reservoir analyses.

Animal ID	Sex	Age[a] (months)	*Mamu*-A*01 status	#CD4[b] d58 p.i.	Intervention[c]	SIV-RNA copies/mL (plasma)[d] d58 p.i.	SIV-RNA copies/mL (plasma)[d] d259 p.i.	PMID[e]
RLm12	F	57	+	540	ART + IL-21	1.07E+05	<60	26551680
ROe12	F	65	-	707	ART + IL-21	1.73E+03	<60	26551680
RBt12	F	57	+	678	ART	6.56E+03	<60	26551680
RGe12	F	67	-	594	ART	3.20E+06	<60	26551680
RJp11	F	81	+	709	ART + IL-21	1.99E+04	<60	26551680
RGv10	F	102	-	379	ART + IL-21	6.94E+05	<60	26551680
RCb12	F	70	+	942	ART	3.38E+03	<60	26551680
RJw12	F	54	-	463	ART	4.54E+05	<60	26551680
RVt10	F	98	+	366	ART + IL-21	6.83E+05	<60	26551680
RTb12	F	71	-	364	ART + IL-21	1.66E+06	<60	26551680
RKg11	F	90	+	1108	ART	2.12E+04	<60	26551680
RPu12	F	57	-	280	ART	2.47E+06	<60	26551680
RKd12	F	68	-	452	ART	1.53E+05	<60	26551680
RPy8	F	120	+	374	ART	3.50E+05	<60	26551680
RHa10	F	114	-	618	ART + IL-21	2.31E+04	<60	26551680

[a]Age in months at time of SIV infection.

[b]#CD4^{+} T-cells per µL of peripheral blood during chronic infection immediately prior to antiretroviral therapy (ART) initiation (d58 post-infection, p.i.) as calculated by flow cytometry in combination with complete blood counts.

[c]At d60 p.i. all rhesus macaques (RMs) began a five-drug ART regimen consisting of raltegravir, emtricitabine, tenofovir, darunavir, and ritonavir. Starting at d60 and d203 p.i. the indicated RMs began 6-dose, weekly cycle of subcutaneous rhesus IL-21-IgFc (IL-21) at 100 µg/kg.

[d]Plasma viral loads ($SIVmac_{239}$ RNA copies/mL) were measured by RT-qPCR during chronic infection (d58 p.i.) and on-ART (d259 p.i.).

[e]Study reference for historical samples by PMID (PubMed identifier).

Table S2. Plasma IL-10 correlates with SIV disease progression independently of other soluble biomarkers of inflammation during chronic infection.

	Independent Variables (d58 p.i.); Spearman's correlation							
	IL-10 pg/mL (plasma)		IP-10 pg/mL (plasma)		log10 SIV-RNA (plasma)		sCD14 µg/mL (plasma)	
Dependent Variable (d58 p.i.)	rho	p_value	rho	p_value	rho	p_value	rho	p_value
$\log_{10}$ SIV-RNA (plasma)	0.7578	0.0016	0.8143	0.0004	1	<0.0001	-0.325	0.237
$\log_{10}$ SIV-DNA (PBMC)	0.7048	0.0043	0.7417	0.0022	0.8722	<0.0001	-0.5219	0.0481
$\log_{10}$ SIV-DNA (RB)	0.7026	0.0064	0.8194	0.0006	0.6982	0.0069	-0.1123	0.7009
$\log_{10}$ SIV-DNA $CD4^+$ (LN)	0.7569	0.0025	0.7407	0.0034	0.8769	<0.0001	-0.5165	0.0615
$\log_{10}$ SIV-DNA T_{CM} $CD4^+$ (LN)	0.7635	0.0022	0.7319	0.004	0.8462	0.0003	-0.4154	0.1413
$\log_{10}$ SIV-DNA T_{EM} $CD4^+$ (LN)	0.7261	0.0044	0.7363	0.0037	0.8901	<0.0001	-0.4681	0.0938
$\log_{10}$ SIV-DNA T_{FH} $CD4^+$ (LN)	0.6007	0.0256	0.6659	0.0113	0.8462	0.0003	-0.3978	0.1602
% Ki-67+ of T_{FH} (LN)	0.8293	0.0002	0.75	0.0019	0.7643	0.0014	-0.1393	0.6205
#$CD4^+$ T-cells (PB)	-0.8204	0.0003	-0.7036	0.0045	-0.7929	0.0007	0.4929	0.0644
IP-10 pg/mL (plasma)	0.849	0.0001	1	<0.0001	0.8143	0.0004	-0.06071	0.8324
	sCD163 ng/mL (plasma)		CRP µg/mL (plasma)		Neopterine ng/mL (plasma)		LPS pg/mL (plasma)	
Dependent Variable (d58 p.i.)	rho	p_value	rho	p_value	rho	p_value	rho	p_value
$\log_{10}$ SIV-RNA (plasma)	0.2571	0.3538	0.05	0.8626	-0.002198	>0.9999	0.08571	0.763
$\log_{10}$ SIV-DNA (PBMC)	0.1966	0.4794	0.0983	0.7264	0.02857	0.9276	0.2216	0.4241
$\log_{10}$ SIV-DNA (RB)	-0.1652	0.5699	0.337	0.2372	0.3912	0.1859	0.1498	0.6069
$\log_{10}$ SIV-DNA $CD4^+$ (LN)	0.3714	0.1918	0.1912	0.5121	-0.05934	0.8438	0.2527	0.3825
$\log_{10}$ SIV-DNA T_{CM} $CD4^+$ (LN)	0.1736	0.5526	0.0989	0.7385	-0.04176	0.8915	0.1648	0.5733
$\log_{10}$ SIV-DNA T_{EM} $CD4^+$ (LN)	0.3187	0.2666	0.06374	0.8319	-0.1209	0.6818	0.1033	0.727
$\log_{10}$ SIV-DNA T_{FH} $CD4^+$ (LN)	0.2659	0.3573	0.433	0.1239	-0.06374	0.8319	0.02857	0.9276
% Ki-67+ of T_{FH} (LN)	0.2393	0.3892	0.3786	0.1649	0.1868	0.5221	0.2036	0.4657
#$CD4^+$ T-cells (PB)	-0.1071	0.7049	-0.3393	0.2161	-0.05495	0.8557	-0.1179	0.6763
IP-10 pg/mL (plasma)	0.2857	0.3012	0.07857	0.7827	0.411	0.1458	0.1321	0.6389

[a]From chronic infection (d58 post-infection, p.i.), virologic and immunologic measures of disease progression (as indicated in each row; see Figure 2A,B,C,D,F,G,H,I and Figure S2A,B) were correlated against plasma IL-10 (Figure 1A), plasma IP-10 (Figure S2B), and plasma viremia (Figure 2A), and markers of inflammation in plasma (as indicated in each column) including: soluble CD14 (sCD14), soluble CD163 (sCD163), C-reactive protein (CRP), neopterin, lipopolysaccharide (LPS).

[b]Statistical analyses were performed using a two-sided non-parametric Spearman correlation. The strength and directionality of the Spearman coefficient (rho) is visually indicated by the overlaid bidirectional, gradient heatmap and significant nominal p-values ($p<0.05$) are highlighted in green.

Table S3. Statistical evaluation of multicollinearity between plasma levels of IL-10, viremia, and IP-10 during chronic infection.

Variables		Regression	Multicollinearity Statistics			
Dependent	Independent	β[a]	Variance Inflation Factor[b]	Tolerance[c]	Eigenvalue[d]	Condition Index[e]
log_{10} SIV-DNA (PBMC) d58 p.i.	IL-10 pg/mL (plasma) d58 p.i.	0.000	2.36	0.42	0.12	5.65
log_{10} SIV-DNA (PBMC) d58 p.i.	log_{10} SIV-RNA copies/mL (plasma) d58 p.i.	0.411	2.66	0.38	0.04	9.26
log_{10} SIV-DNA (PBMC) d58 p.i.	IP-10 pg/mL (plasma) d58 p.i.	0.001	3.54	0.28	0.01	19.76
log_{10} SIV-DNA (RB) d58 p.i.	IL-10 pg/mL (plasma) d58 p.i.	-0.004	2.18	0.46	0.11	6.03
log_{10} SIV-DNA (RB) d58 p.i.	log_{10} SIV-RNA copies/mL (plasma) d58 p.i.	0.009	2.64	0.38	0.04	9.3
log_{10} SIV-DNA (RB) d58 p.i.	IP-10 pg/mL (plasma) d58 p.i.	0.003	3.34	0.3	0.01	19.48
log_{10} SIV-DNA $CD4^+$ (LN) d58 p.i.	IL-10 pg/mL (plasma) d58 p.i.	-0.004	2.37	0.42	0.13	5.43
log_{10} SIV-DNA $CD4^+$ (LN) d58 p.i.	log_{10} SIV-RNA copies/mL (plasma) d58 p.i.	0.355	2.77	0.36	0.05	9
log_{10} SIV-DNA $CD4^+$ (LN) d58 p.i.	IP-10 pg/mL (plasma) d58 p.i.	0.001	3.81	0.26	0.01	19.78
log_{10} SIV-DNA T_{CM} $CD4^+$ (LN) d58 p.i.	IL-10 pg/mL (plasma) d58 p.i.	0.001	2.37	0.42	0.13	5.43
log_{10} SIV-DNA T_{CM} $CD4^+$ (LN) d58 p.i.	log_{10} SIV-RNA copies/mL (plasma) d58 p.i.	0.461	2.77	0.36	0.05	9
log_{10} SIV-DNA T_{CM} $CD4^+$ (LN) d58 p.i.	IP-10 pg/mL (plasma) d58 p.i.	0.000	3.81	0.26	0.01	19.78
log_{10} SIV-DNA T_{EM} $CD4^+$ (LN) d58 p.i.	IL-10 pg/mL (plasma) d58 p.i.	-0.001	2.37	0.42	0.13	5.43
log_{10} SIV-DNA T_{EM} $CD4^+$ (LN) d58 p.i.	log_{10} SIV-RNA copies/mL (plasma) d58 p.i.	0.533	2.77	0.36	0.05	9
log_{10} SIV-DNA T_{EM} $CD4^+$ (LN) d58 p.i.	IP-10 pg/mL (plasma) d58 p.i.	0.000	3.81	0.26	0.01	19.78
log_{10} SIV-DNA T_{FH} $CD4^+$ (LN) d58 p.i.	IL-10 pg/mL (plasma) d58 p.i.	-0.010	2.37	0.42	0.13	5.43
log_{10} SIV-DNA T_{FH} $CD4^+$ (LN) d58 p.i.	log_{10} SIV-RNA copies/mL (plasma) d58 p.i.	0.539	2.77	0.36	0.05	9
log_{10} SIV-DNA T_{FH} $CD4^+$ (LN) d58 p.i.	IP-10 pg/mL (plasma) d58 p.i.	0.001	3.81	0.26	0.01	19.78
% Ki-67+ of T_{FH} (LN) d58 p.i.	IL-10 pg/mL (plasma) d58 p.i.	0.348	2.36	0.42	0.12	5.65
% Ki-67+ of T_{FH} (LN) d58 p.i.	log_{10} SIV-RNA copies/mL (plasma) d58 p.i.	2.743	2.66	0.38	0.04	9.26
% Ki-67+ of T_{FH} (LN) d58 p.i.	IP-10 pg/mL (plasma) d58 p.i.	0.016	3.54	0.28	0.01	19.76
# $CD4^+$ T-cells (PB) d58 p.i.	IL-10 pg/mL (plasma) d58 p.i.	-6.961	2.36	0.42	0.12	5.65
# $CD4^+$ T-cells (PB) d58 p.i.	log_{10} SIV-RNA copies/mL (plasma) d58 p.i.	-116.000	2.66	0.38	0.04	9.26
# $CD4^+$ T-cells (PB) d58 p.i.	IP-10 pg/mL (plasma) d58 p.i.	-0.056	3.54	0.28	0.01	19.76

[a]β is the regression coefficient for the independent variable.

[b]The Variance Inflation Factor (VIF) measures the inflation in the variances of the dependent variable estimates due to collinearity between the independent variables. Typically, a VIF of 1 indicates no collinearity between independent variables; VIFs exceeding 4 warrant further investigation; and VIFs exceeding 10 indicate severe collinearity.

[c]Tolerance is a measure of the percentage of variance that cannot be accounted for by the other predictors and should not lower than 0.10. Defined as $1 - R^2$.

[d]The Eigenvalue is the variance of linear combinations upon decomposing the correlation matrix.

[e]The Condition Index gives the degree of multicollinearity in a regression matrix. Multicollinearity is regarded as a problem if the Condition Index exceeds 30 and more than 2 independent variables have Eigenvalues of greater than 0.50.

Table S4. Animals included for LN IHC and DNAscope analyses.

Animal ID	Analysis[a]	Sex	Age[b] (months)	*Mamu*-A*01[c] status	# CD4[d] chronic	viral load (copies/mL)[e] chronic	ART	Timing of ART collection[f]	PMID[g]
ZI26	IHC/DNAscope	M	55	ND	559	5.00E+06	190	UD (uninfected, chronic, ART)	N/A
ZI60	IHC/DNAscope	M	53	ND	954	1.50E+07	390	UD (uninfected, chronic, ART)	N/A
DE9P	IHC/DNAscope	M	54	ND	658	8.30E+06	40	UD (uninfected, chronic, ART)	N/A
DEMD	IHC/DNAscope	M	171	ND	486	8.50E+07	N/A	UD (uninfected, chronic)	N/A
DCCP	IHC	M	35[Ψ]	+	976	8.10E+06	<30	UD (uninfected, ART)	25182644
DCLJ	IHC	M	39[Ψ]	+	882	3.70E+05	<30	UD (uninfected, ART)	25182644
DCHV	IHC	M	44[Ψ]	-	2946	3.50E+06	<30	UD (uninfected, ART)	26711758
DCW9	IHC	M	41[Ψ]	-	906	1.10E+07	70	UD (uninfected, ART)	26711758
GRH	IHC	F	64[Ψ]	-	1228	2.10E+06	<30	UD (uninfected, ART)	26711758
GRB	IHC	F	64[Ψ]	-	1633	7.00E+06	100	UD (uninfected, ART)	N/A
ZK30	IHC	M	32	-	1900	N/A	N/A	N/A (uninfected)	N/A
DF94	IHC	M	35	-	2579	N/A	N/A	N/A (uninfected)	N/A
ZL61	IHC	M	28	-	2452	N/A	N/A	N/A (uninfected)	N/A
ZL65	IHC	M	28	-	1920	N/A	N/A	N/A (uninfected)	N/A
ZL23	IHC	F	28	-	1211	N/A	N/A	N/A (uninfected)	N/A
MBZ	IHC	F	47	-	2710	N/A	N/A	N/A (uninfected)	N/A
DFEN	IHC	M	50	-	980	N/A	N/A	N/A (uninfected)	N/A
DEZZ	IHC	M	50	-	1356	N/A	N/A	N/A (uninfected)	N/A
DF36	IHC	M	50	-	1539	N/A	N/A	N/A (uninfected)	N/A
DEGL	IHC	M	46	-	1185	N/A	N/A	N/A (uninfected)	N/A
ZJ14	IHC	F	47	-	1211	N/A	N/A	N/A (uninfected)	N/A
ZJ54	IHC	F	44	-	611	N/A	N/A	N/A (uninfected)	N/A
ZJ33	IHC	F	44	+	1591	N/A	N/A	N/A (uninfected)	N/A
DCBB	IHC	M	64	-	ND	N/A	N/A	N/A (uninfected)	26282376
RKd12	IHC	F	68	-	452	1.53E+05	N/A	d58	26551680
RGv10	DNAscope/IHC	F	102	-	379	6.94E+05	<30	d58/203	26551680
RJp11	DNAscope/IHC	F	81	+	709	1.99E+04	N/A	d58	26551680
RBt12	DNAscope/IHC	F	57	+	678	6.56E+03	<30	d58/197	26551680
RCb12	DNAscope/IHC	F	70	+	942	3.38E+03	<30	d58/240	26551680
RPy8	DNAscope	F	120	+	374	3.50E+05	<30	d58/203	26551680
RTb12	DNAscope/IHC	F	71	-	364	1.66E+06	37	d59/198	26551680
RHa10	DNAscope	F	114	-	618	2.31E+04	N/A	d58	26551680

[a]Indicates whether rhesus macaques (RMs) were utilized for IHC (immunohistochemistry) or DNAscope analyses.

[b]RM age, in months, at either SIV infection or sample collection (if not experimentally infected); [Ψ] annotation indicates age at antiretroviral therapy (ART) initiation.

[c]*Mamu*-A*01 status: + (positive), – (negative), or ND (not determined).

[d]nadir count of $CD4^+$ T-cells per µL of peripheral blood (PB) during chronic infection.

[e]viral load ($SIVmac_{239}$ RNA copies per mL) in plasma during chronic infection, on-ART, or N/A (not applicable)

[f]Indicates the relative timing of lymph node (LN) collections in days post-infection, UD (undetermined) if not known, or N/A (not applicable) if they did not receive ART. Per each animal listed as UD or NA, the study phase used for analysis is provided.

[g]Study reference for historical samples by PMID (PubMed identifier) or N/A (not applicable).

The following RMs were ART-treated: ZI26, ZI60, DE9P, DCCP, DCLJ, DCHV, DCW9, GRH, GRB, RKd12, RGv10, RJp11, RBt12, RCb12, RPy8, RTb12, and RHa10. The following animals were not experimentally SIV-infected: ZK30, DF94, ZL61 ZL65, ZL23, MBZ, DFEN, DEZZ, DF36, DEGL, ZJ14, ZJ54, ZJ33, and DCBB.

Table S5. Plasma IL-10 predicts SIV reservoir content independent of other soluble biomarkers during suppressive ART.

	Independent Variables (d58 p.i.); Spearman's correlation							
	IL-10 pg/mL (plasma)		IP-10 pg/mL (plasma)		log10 SIV-RNA (plasma)		sCD14 μg/mL (plasma)	
Dependent Variable (d259 p.i.)	rho	p_value	rho	p_value	rho	p_value	rho	p_value
log_{10} SIV-DNA (PBMC)	0.5934	0.0218	0.6071	0.0186	0.7821	0.0009	-0.5286	0.0454
log_{10} SIV-DNA (RB)	0.5648	0.0383	0.6747	0.01	0.4989	0.0721	0.2879	0.3176
% T_{FH} of $CD4^+$ T-cells (LN)	0.6881	0.0058	0.4929	0.0644	0.6786	0.0068	-0.5214	0.0488
IUPM $CD4^+$ (LN)	0.8	0.3333	0.8	0.3333	1	0.0833	0.6	0.4167
	sCD163 ng/mL (plasma)		CRP μg/mL (plasma)		Neopterine ng/mL (plasma)		LPS pg/mL (plasma)	
Dependent Variable (d259 p.i.)	rho	p_value	rho	p_value	rho	p_value	rho	p_value
log_{10} SIV-DNA (PBMC)	-0.02143	0.9438	0.09286	0.7435	-0.06374	0.8319	0.2036	0.4657
log_{10} SIV-DNA (RB)	0.1033	0.727	-0.2088	0.4731	0.6209	0.0268	-0.05055	0.8676
% T_{FH} of $CD4^+$ T-cells (LN)	-0.2679	0.3335	0.25	0.3678	-0.04615	0.8796	0.4929	0.0644
IUPM $CD4^+$ (LN)	0.8	0.3333	-0.4	0.75	0	>0.9999	-0.4	0.75

[a]Virologic and immunologic measures of SIV reservoir content during antiretroviral therapy (ART; d259 post-infection, p.i.; as indicated in each row; see Figure 4A,B,C,D) were correlated against parameters in plasma during chronic infection (d58 p.i.; as indicated in each column) including IL-10 (Figure 1A), IP-10 (Figure S2B), viremia (Figure 2A), and soluble markers of inflammation including soluble CD14 (sCD14), soluble CD163 (sCD163), C-reactive protein (CRP), neopterin, lipopolysaccharide (LPS).

[b]Statistical analyses were performed using a two-sided non-parametric Spearman correlation. The strength and directionality of the Spearman coefficient (rho) is visually indicated by the overlaid bidirectional, gradient heatmap and significant nominal p-values ($p<0.05$) are highlighted in green.

Table S6. Characteristics of rhesus macaques utilized for IL-10 neutralization.

Animal ID	Sex	Age (months)	*Mamu*-A*01 status	#CD4[b] d35 p.i.	Intervention[c]	SIV-RNA copies/mL (plasma)[d] d35 p.i.	d209 p.i.
RRs15	M	46	+	1940	ART + αIL10 mAb	5.30E+04	<15
RSr15	M	46	+	390	ART + αIL10 mAb	2.60E+05	<15
RQv15	M	45	-	2112	ART + αIL10 mAb	3.80E+05	<15
RNw15	M	45	-	958	ART + αIL10 mAb	9.70E+04	<15
RPz15	M	44	-	1305	ART + αIL10 mAb	4.30E+04	<15
RBf16	M	43	-	1375	ART + αIL10 mAb	4.00E+06	40

[a]Age in months at time of SIV infection.
[b]#CD4^{+} T-cells per μL of peripheral blood during chronic infection immediately prior to antiretroviral therapy (ART) initiation (d35 post-infection, p.i.) as calculated by flow cytometry in combination with complete blood counts.
[c]At d35 p.i. all rhesus macaques (RMs) began a three-drug ART regimen consisting of dolutegravir, emtricitabine, and tenofovir. All RMs were intravenously administered an anti-IL-10 monoclonal antibody (αIL-10 mAb) on d211 and d238 p.i. at 10 mg/kg and 20 mg/kg, respectively.
[d]Plasma viral loads (SIVmac$_{239}$ RNA copies/mL) were measured by RT-qPCR during early chronic infection (d35 p.i.) and at the pre-treatment baseline on-ART (d209 p.i.).

Supplemental Methods

Flow cytometry

The following 13-parameter mAb panel was used in our IL-10 characterization cohort: anti-CD3–APC–Cy7 (clone SP34-2; 5 µL; cat. 557757), anti-CD95–PE–Cy5 (clone DX2; 10 µL; cat. 559773), anti-CD28–PE-CF594 (clone CD28.2; 5 µL; cat. 562296), anti–Ki-67–Alexa Fluor 700 (clone B56; 5 µL; cat. 561277), and anti-CCR7–PE–Cy7 (clone 3D12; 7.5 µL; cat. 557648) all from BD Biosciences; anti-CD4–BV605 (clone OKT4; 5 µL; cat. 317438), anti-PD1–BV421 (clone EH12.2H7; 5 µL; cat. 329920), and anti-CD20-PerCP-Cy5.5 (clone 2H7; 5 µL; cat. 302326) all from Biolegend; anti-CD8–Qdot705 (clone 3B5; 1 µL; cat. Q10059), anti-CXCR5-PE (clone MU5UBEE; 5 µL; cat. 12-9185-42), and LIVE/DEAD Fixable Aqua Dead Cell Stain (2 µL of 1:10 dilution; cat. L34957) all from ThermoFisher; and anti-α4β7-APC (clone A4B7R1; 5 µL; RRID AB_2819257) from the Nonhuman Primate Reagent Resource. The following 17-parameter mAb panel was used in our IL-10 neutralization cohort: anti-CCR7–PE–Cy7 (clone 3D12; 7.5 µL; cat. 557648), anti-CTLA-4-BV421 (clone BNI3; 10 µL; cat. 562743), anti-CD3-BUV395 (clone SP34-2; 5 µL; cat. 564117), anti-CD8-BUV496 (clone RPA-T8; 5 µL; cat. 612942), and anti-CD28-BUV737 (clone CD28.2; 5 µL; cat. 612815) all from BD Biosciences; anti-T-bet-PE/Dazzle594 (clone 4B10; 5 µL; cat. 644828), anti-FoxP3-AF647 (clone 150D; 5 µL; cat. 320014), anti-CD4-APC-Cy7 (clone OKT4; 5 µL; cat. 317418), anti-CD25-BV711 (clone BC96; 5 µL; cat. 302636), anti-CD95-BV605 (clone DX2; 5 µL; cat. 305628), anti-HLA-DR-BV650 (clone L243; 5 µL; cat. 307650), and anti-PD-1-BV785 (clone EH12.2H7; 5 µL; cat. 329930) all from Biolegend; anti-CD38-FITC (clone AT-1; 5 µL; cat. 60131FI) from STEMCELL

Technologies; and anti-CXCR5-PE (clone MU5UBEE; 5 μL; cat. 12-9185-42), anti-CD127-PE-Cy5 (clone eBioRDR5; 5 μL; cat. 15-1278-42), and LIVE/DEAD Fixable Aqua Dead Cell Stain (2 μL of 1:10 dilution; cat. L34957) all from ThermoFisher.

Quantitative Viral Outgrowth Assay (QVOA)

As previously described (1, 2), $CD4^+$ cells from cryo-preserved LN were isolated in by positive selection using CD4 Microbeads (cat. 130-091-102; Miltenyi Biotec). Isolated $CD4^+$ cells were stimulated for up to 16 hours at 37°C with IL-2 (20 ng/mL; cat. 21-8029-U010; Tonbo Biosciences), anti-CD2 mAb (clone RPA-2.10, 5 μg/mL; cat. 300202; Biolegend), and anti-CD28 mAb (clone CD28.2, 5 μg/mL; cat. 556620; BD Pharmingen) in a 96-well polystyrene plated coated with anti-CD3 mAbs (clone SP34-2, 1 μg/well; cat. 551916; BD Biosciences). Stimulated $CD4^+$ cells were co-cultured at a 1:1 ratio with the 174xCEM cell line (3), which is a fusion between the human CEM T-cell line with human 721.174 B-cell line (NIH AIDS Research and Reference Reagent Program; cat. ARP-272) in 4 serial dilutions ranging from $0.1\text{-}1\times10^6$ cells per well with 2-4 replicates per dilution. Information on the 174xCEM cell line gender is not available and the cell line was not authenticated. The cells were cultured in complete RPMI 1640 with 4 mM L-glutamine supplemented with 10% heat-inactivated fetal bovine serum, penicillin (50 U/ml), streptomycin (50 μg/ml), and IL-2 (100 U/ml; all from Gemini Bio). Cultured cells were fed with fresh medium weekly, and were harvested and analyzed on days 9, 16, and 25 (**Table S1**; RJw12, RPu12, RKd12, and RPy8; n=4). Well positivity was determined by a greater than 10-fold amplification of SIV-RNA (copies/mL) in the supernatant by qRT-PCR and/or by p27 (clone 55-2F12; cat. ARP-1610, HIV Reagent Program) expression via flow cytometry (4). The frequencies

of infected cells were determined by the maximum likelihood method (5) utilizing IUPMStats (6) and expressed in terms of infectious units per million (IUPM) $CD4^+$ cells.

Immunohistochemistry

IL-10 IHC in formalin-fixed, paraffin-embedded (FFPE) LNs was essentially performed as previously described (7) for mouse IL-10 (1-2 μg/mL; clone E-10, Santa Cruz Biotechnology). In brief, IHC was performed using a biotin-free polymer approach (Mouse Polink-2, Golden Bridge International, Inc.) on 5 μm tissue sections mounted on glass slides, which were dewaxed and rehydrated with double-distilled H_2O. Antigen retrieval was performed by heating slides in 0.1M Tris-HCL pH 8.6 in a pressure cooker set at 122-125ºC for 30 sec. After cooling and rinsing in ddH_2O, slides were incubated in blocking buffer (TBS with 0.05% Tween-20 and 0.5% casein) for 10 min, followed by an endogenous peroxidase blocking solution using 1.5% (v/v) H_2O_2 in TBS pH 7.4 for 10 min. Anti-IL-10 was diluted at 1:100-200 in blocking buffer and incubated overnight at room temperature. Tissue sections were washed and detected using the Mouse Polink-2 staining system (Golden Bridge International, Inc) according to manufacturer's recommendations. Sections were developed with Impact™ 3,3'-diaminobenzidine (Vector Laboratories), counterstained with hematoxylin and mounted in Permount (Fisher Scientific). All stained slides were scanned at high magnification (200-400x) using the Aperio® AT2 System (Leica Biosystems) yielding high-resolution data for the entire tissue section. Representative high magnification (400x) images were acquired from these whole tissue scans. Representative regions of interest (ROIs; 0.04 mm^2) were identified and high-resolution images extracted from these whole-tissue scans. The percentage of the area of the LN anatomic BCF and TCZ compartments was quantified using Photoshop (CS5 and CS6; Adobe Systems), Noiseware 5 (Imagenomic) for noise reduction, and Fovea Pro 4

(Reindeer Graphics) image analysis tools. High resolution ROI images were analyzed by segmenting the positive area with color channel thresholding in Photoshop and measuring the positive area percentage with Fovea.

SIV RNA chromagen in situ hybridization

RNAscope was performed on formaldehyde-fixed, paraffin-embedded tissue sections (5 μm) according to our previously published protocol (8) with the following minor modifications: heat-induced epitope retrieval was performed by boiling slides in 1x RNAscope target retrieval (ACDBio, cat. 322000) for 30 min, followed by incubation at 40°C with a 1:5 to 1:10 dilution of protease III (ACDBio, cat. 322337) in 1x PBS for 20 min. Slides were incubated with the anti-sense viral RNA target probe $SIVmac_{239}$ (ACDBio, cat. 312811) for 2 hr at 40°C and amplification was performed with RNAscope 2.5 HD Detection Red kit (ACDBio, cat. 322360; ACD) according to manufacturer's instructions, with the exception that wash steps were performed with 0.5X RNAscope wash buffer (ACDBio, cat. 310091). The resulting signal was developed with Warp Red chromogen (Biocare Medical, cat. WR806M). Slides were counterstained with CAT hematoxylin (Biocare Medical, cat. CATHE-GL), mounted with Clearmount (EMS, cat. 17885-15) until dry, cover slipped using Permount (Fisher Scientific, cat. SP15-100), and scanned at 40x magnification on an Aperio AT2 (Leica Biosystems). RNAscope images were analyzed to quantify the total number of $vRNA^+$ cells per 10^6 total cells and using the ISH module (v2.2) within HALO software (Indica Labs v3.0.311.405). Since probe size is controlled by several malleable experimental procedures, module settings were established on concomitantly-ran, acutely-infected SIV^+ control slides to determine the probe area (min/mean/max) of ≥10 identifiable SIV virions, which correspond to two copies of vRNA. The vRNA minimum probe spot size within the analysis

module was therefore selected to include cells containing ≥3 copies of vRNA to exclude the potential detection of one integrated copy of positive sense vDNA. Location within the lymph node was determined by staining sequential slides with rabbit anti-CD4 (clone EPR6855; cat. Ab133616; Abcam) and mouse anti-CD20 (clone L26; cat. CM004C; Biocare), and separating the tissue into either T-cell zone, B-cell follicle, or medullary cord compartments using a random forest algorithm for the classifier module in HALO. Manual curation was performed on each sample to correct for false positives or negatives.

SIV DNAscope with multiplex immunofluorescence

Next-generation DNAscope *in situ* hybridization and multiplex immunofluorescence staining was performed on 5 μm thick sections from FFPE lymph node biopsies from chronically SIV infected animals before and during ART (**Table S4**) (1). DNAscope was performed as previously described (8) with some modifications. Briefly, slides were deparaffinized by heating at 60°C for 1 hr, followed by two 5 min xylene incubations, two 1 min 100% ethanol incubations and then slides were kept in double distilled water before heat-induced epitope retrieval by boiling with the RNAscope Target Retrieval Buffer (ACDBio, cat. 322000) for 30 min. Slides were then rinsed twice in double distilled water and tissues were kept wet throughout the rest of the protocol. Slides were then treated with ACDBio Protease III diluted 1:10 in cold PBS for 20 min at 40°C and then washed twice with distilled water. Slides were incubated for 10 min at room temperature with 3% hydrogen peroxide diluted in PBS to inactivate endogenous peroxidases. The $SIVmac_{239}$ sense probe (ACDbio, cat. 314071) was added to the tissue for a 14 hr incubation at 40°C in a HybEz oven. The RNAscope 2.5 HD Brown kit (ACDBio, cat. 322310) was used to amplify vDNA signal according to manufacturer's recommendations, with the exception that wash steps were performed

with 0.5X RNAscope wash buffer (ACDBio, cat. 310091). In addition, after Amp 5 and 6, slides were washed with 1X TBS-Tween-20 (0.05% v/v) instead of RNAscope wash buffer. Viral DNA fluorescence signal was developed using CF568 conjugated tyramide reagent (Biotium; cat. 92173). Immunofluorescence antibody staining was performed using a mouse anti-IL-10 antibody (Santa Cruz, cat. sc-8438), a rabbit anti-pSTAT3 antibody (Cell Signaling, cat. 9145) and a goat polyclonal anti-CD20 antibody (Invitrogen, cat. PA1-9024), followed by incubation with the following donkey secondary antibodies: AF647 conjugated donkey anti-mouse (Thermo Fisher, cat. A31571), AF755 conjugated donkey anti-rabbit (Thermo Fisher Scientific, cat. SA5-10043) and AF488 conjugated donkey anti-goat (Thermo Fisher, cat. A11055). Tissues were counterstained with DAPI (Invitrogen, cat. D1306; 500 ng/mL) and cover slipped with #1.5 GOLD SEAL cover glass (EMS, cat. 63791-10) using Prolong® Gold reagent (ThermoFisher; cat. P36930). To quantify the number of vDNA^{+}, IL-10^{+} and/or pSTAT3^{+} cells on multiplex stained tissues, whole-slide high-resolution fluorescent scans were performed using a Plan-Apochromat 20X objective (NA 0.80) using the Zeiss AxioScan Z.1 slide scanner. DAPI, AF488, AF568, Cy5 (For AF647) and Cy7 (For AF755) channels were used to acquire images. The exposure time for image acquisition was between 4 and 300 ms. Multi-spectral images were analyzed using the HALO 3.0 platform (Indica Labs) using the following analysis methods: For SIV vDNA, cells with clear punctate dots within the nucleus were quantified using the module FISH v1.1. Thresholds for spot size and intensity were standardized against the vDNA signal in 3D8 cells, which has at least one integrated provirus per cell. Total cell count per tissue was also performed using this module. For the quantification of the total number of pSTAT3^{+} and IL-10^{+} cells per tissue, the HighPlex FL v3.0.3 module was used. The Nearest Neighbor and Proximity Analysis were performed with the Spatial Analysis plot function in HALO, using the object data derived

from the previous individual analyses discussed above. Manual curation was performed to confirm the accurate quantification of vDNA$^+$ cells and their individual IL-10 and pSTAT3 status.

Peptide Stimulations

Samples were immunophenotyped with the following rhesus-reactive mAbs: anti-IL-2-BB700 (clone MQ1-17H12; 5 µL; cat. 566405), anti-CD45-BV480 (clone D058-1283; 5 µL; cat. 566152), anti-CD4-BV650 (clone L200; 5 µL; cat. 563737), anti-IFNγ-BV711 (clone B27; 5 µL; cat. 564039); anti-CD3-BUV395 (clone SP34-2; 5 µL; cat. 564117), and anti-CD8-BUV563 (clone RPA-T8; 5 µL; cat. 612914) all from BD Biosciences; anti-PD-1-BV421 (clone EH12.2H7; 5 µL; cat. 329920) and anti-CD95-BV605 (clone DX2; 5 µL; cat. 305628) from Biolegend; and anti-TNFα-PE-Cy7 (clone MAB11; 2.5 µL; cat. 25-7349-82) and LIVE/DEAD Fixable Far Red (1 µL; cat. L34976) from ThermoFisher.

Supplemental Methods References

1. Micci L, et al. Interleukin-21 combined with ART reduces inflammation and viral reservoir in SIV-infected macaques. *J Clin Invest.* 2015;125(12):4497-513.
2. Laird GM, et al. Rapid quantification of the latent reservoir for HIV-1 using a viral outgrowth assay. *PLoS Pathog.* 2013;9(5):e1003398.
3. Salter RD, et al. Genes regulating HLA class I antigen expression in T-B lymphoblast hybrids. *Immunogenetics.* 1985;21(3):235-46.
4. Fukazawa Y, et al. B cell follicle sanctuary permits persistent productive simian immunodeficiency virus infection in elite controllers. *Nat Med.* 2015;21(2):132-9.
5. Myers LE, et al. Dilution assay statistics. *J Clin Microbiol.* 1994;32(3):732-9.

6. Rosenbloom DI, et al. Designing and interpreting limiting dilution assays: general principles and applications to the latent reservoir for human immunodeficiency virus-1. *Open Forum Infect Dis.* 2015;2(4):ofv123.
7. Tabb B, et al. Reduced inflammation and lymphoid tissue immunopathology in rhesus macaques receiving anti-tumor necrosis factor treatment during primary simian immunodeficiency virus infection. *J Infect Dis.* 2013;207(6):880-92.
8. Deleage C, et al. Defining HIV and SIV Reservoirs in Lymphoid Tissues. *Pathog Immun.* 2016;1(1):68-106.
